# Characterization of the membrane interactions of phospholipase Cγ reveals key features of the active enzyme

**DOI:** 10.1126/sciadv.abp9688

**Published:** 2022-06-24

**Authors:** Kyle I. P. Le Huray, Tom D. Bunney, Nikos Pinotsis, Antreas C. Kalli, Matilda Katan

**Affiliations:** 1 Astbury Centre for Structural Molecular Biology, Faculty of Biological Sciences, University of Leeds, Leeds LS2 9JT, UK.; 2 School of Molecular and Cellular Biology, Faculty of Biological Sciences, University of Leeds, Leeds, LS2 9JT UK.; 3 Leeds Institute of Cardiovascular and Metabolic Medicine, School of Medicine, University of Leeds, Leeds LS2 9JT, UK.; 4 Institute of Structural and Molecular Biology, Division of Biosciences, University College London, Gower St., London WC1E 6BT, UK.; 5 Institute of Structural and Molecular Biology, Birkbeck College, London, WC1E 6BT, UK.

## Abstract

PLCγ enzymes are autoinhibited in resting cells and form key components of intracellular signaling that are also linked to disease development. Insights into physiological and aberrant activation of PLCγ require understanding of an active, membrane-bound form, which can hydrolyze inositol-lipid substrates. Here, we demonstrate that PLCγ1 cannot bind membranes unless the autoinhibition is disrupted. Through extensive molecular dynamics simulations and experimental evidence, we characterize membrane binding by the catalytic core domains and reveal previously unknown sites of lipid interaction. The identified sites act in synergy, overlap with autoinhibitory interfaces, and are shown to be critical for the phospholipase activity in cells. This work provides direct evidence that PLCγ1 is inhibited through obstruction of its membrane-binding surfaces by the regulatory region and that activation must shift PLCγ1 to a conformation competent for membrane binding. Knowledge of the critical sites of membrane interaction extends the mechanistic framework for activation, dysregulation, and therapeutic intervention.

## Teaser

A combination of molecular simulations and experiments is used to determine how PLCγ, an important protein, binds to membranes.

## INTRODUCTION

Receptors for a wide range of external stimuli, such as neurotransmitters, agonists for immune cell receptors, hormones, and growth factors, activate core signaling modules, including phospholipase C (PLC) enzymes (*1*). PLC enzymes catalyze the hydrolysis of inositol lipids, mainly phosphatidylinositol 4,5-bisphosphate (PIP_2_) to second messengers inositol 1,4,5-trisphosphate (IP_3_) and diacylglycerol. In addition, PLC enzymes can affect the function of a range of PIP_2_-binding proteins by changing local concentrations of PIP_2_ (*2*). Numerous studies of individual PLCs, including the 16 isoforms in humans, revealed a rich repertoire of PLC functions in different physiological contexts. Furthermore, genetic studies have uncovered many mutated and rare variants of PLC enzymes, particularly in the PLCγ family, and their link to human disease development (*1*, *3*). Specifically, variants of PLCγ1 and PLCγ2 have been linked to cancer, complex immune disorders, inflammation (further implicated in other diseases), and Alzheimer’s and related neurodegenerative diseases [see (*4*–*11*) for the major, comprehensive discoveries].

The understanding of the molecular mechanisms of regulation and dysregulation of PLC enzymes has been facilitated by studies of individual isoforms and their complexes with regulatory proteins (*12*–*20*). An emerging general concept of PLC activation is centered on intramolecular interactions that maintain PLCs in their inactive form, also referred to as autoinhibition, that becomes released in the process of activation in stimulated cells. All structures reported so far seem to represent autoinhibited forms of PLCs. Nevertheless, it is clear that an active form of PLC enzymes must interact with the membrane for the efficient binding and hydrolysis of inositol lipid substrates. The catalytic activity in all PLCs is encapsulated within the TIM (triose-phosphate isomerase) barrel fold structure, which, in most isoforms, together with a PH domain, C2 domain, and EF hands, forms a common PLC core (*1*).

Comprehensive analyses of some of the PLC enzymes also identified specific features involved in regulation of a particular family and suggested that membrane interactions could be quite complex, with multiple roles. In this respect, members of the PLCβ family have been studied most extensively; in addition to structural studies (*13*–*17*), regulation of these enzymes has been analyzed in the presence of different model membranes in vitro (*21*–*25*). In its basal state, PLCβ is autoinhibited by the XY-linker from the catalytic TIM barrel and by elements from the C-terminal domain, representing a unique feature of PLCβ enzymes. Activation of PLCβ involves allosteric conformational changes resulting from the binding of Gαq or Gβγ subunits of heterotrimeric G proteins. PLCβ interactions with the membrane are also likely to contribute to displacement of autoinhibitory constraints, resulting in interfacial activation.

Compared to PLCβ, insights that revealed autoinhibitory interactions in the PLCγ family have been obtained relatively recently from structures of PLCγ1 (*18*, *19*). PLCγ enzymes are characterized by an array of domains, referred to as “γ-specific array (γSA)” or as “regulatory region,” encompassing the sPH, nSH2, cSH2, and SH3 domains. The autoinhibitory interfaces lock the γSA on top of the PLC core. The structures also suggest mechanisms of activation via physiologically relevant phosphorylation of PLCγ by tyrosine kinases and by gain-of-function mutations discovered across diverse pathologies. The location of mutated amino acids mapped to the structure of PLCγ1 reveals that many, including most of the hotspot alterations, are likely to directly release autoinhibition (*18*, *19*). With respect to the PLC core, it has been established that although the same domains (nPH, EF-hand, catalytic TIM barrel, and C2 domains) are present in other families, their function varies, except for the catalytic domain (*1*). Despite initial studies suggesting the role of the PLCγ1 nPH domain in selective binding of PI (3,4,5)P_3_ and the involvement of PLCγ2 C2 domain in Ca^2+^-dependent translocation to the membrane, the overall, direct information about membrane interactions and their possible roles are lacking for PLCγ enzymes (*26*, *27*). Furthermore, as is the case for other PLCs, the active form of PLCγ has not been structurally defined, and this will be required to understand fully the activation processes and different mechanistic classes of disease-linked mutations.

Insights into an active form of PLC enzymes, including PLCγ, are closely linked to understanding of protein/membrane interactions, which bring the enzymes to the membrane proximity, and interactions that position the TIM barrel domain in a productive orientation for catalysis. On the basis of the current evidence, it is likely that an active form can be captured for structural studies only in the presence of model membranes that could be required to stabilize this form or, in some instances, to generate an active form through a membrane-dependent process. These types of protein/membrane complexes still present a challenge for most current structural approaches. Molecular dynamics (MD simulations) can fill this gap, by providing realistic insights into the interactions of proteins with model membranes (*28*–*30*). In particular, MD simulations have been used to predict membrane-binding interfaces, identify lipid binding sites and membrane-inserting regions for key signaling proteins including PTEN, KRAS, bacterial phospholipases, and pleckstrin homology domains (*31*–*35*).

In this study, we used these computational methods to examine the membrane interactions of PLCγ1, in combination with crystallography, direct assessments of protein binding to liposomes, and measurements of PLC activity in vitro and in cells. We identified surfaces and key residues involved in extensive interactions with anionic lipids, especially phosphoinositides, and those inserted into the hydrophobic membrane core. We have also shown the importance of a number of these key residues for overall membrane binding, productive substrate recognition, and catalysis. Furthermore, we found that the regions involved in membrane binding largely overlap with autoinhibitory interfaces on the PLC core. These findings, which identify previously unknown features of membrane interactions of an active enzyme, shed new light on regulation of PLCγ enzymes.

## RESULTS

### Structure of autoinhibited PLCγ1 in complex with IP_3_ and Ca^2+^

**Fig. 1.**
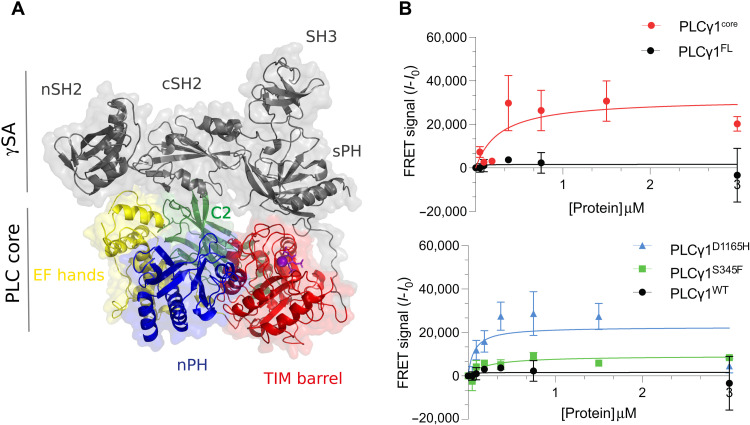
Structure and liposome binding properties of PLCγ1 variants. (**A**) The crystal structure of the IP_3_/PLCγ1 complex in an autoinhibited form (PDB ID: 7Z3J) with the domains of the PLCγ1-specific array (γSA) shown in gray and the domains in the PLC core colored individually (TIM barrel, red; nPH, blue; EF hands, yellow; C2, green); calcium ion and IP_3_ in the active site are shown in purple. (**B**) Comparison of different variants of PLCγ1 for interaction with liposomes. The full-length (FL) and PLC core (core) are shown in the top, and the FL variants with indicated mutations and the WT are shown in the bottom.

Recent structural insights have revealed PLCγ1 in its autoinhibited state (*18*, *19*), which is characterized by an inhibitory interface between the catalytic PLC core domains and the regulatory γSA. To gain further structural insights, we have obtained a new structure of PLCγ1, resolved to 2.0 Å, in its autoinhibited form and in the complex with IP_3_ that has not been previously described for this PLC (Fig. 1A, fig. S1, and table S1). Our structure [Protein Data Bank (PDB) ID 7Z3J] reveals that the active site remains accessible to inositol phosphate, despite autoinhibition. The geometry of the active site is largely conserved compared to the structure of the related PLCδ1—coordination of phosphate 1 of IP_3_ involves H335, H380, and Ca^2+^, the position 4 and 5 phosphates engage in electrostatic interactions, including interaction of the position 4 phosphate with K462 and R1010, and the 2-hydroxyl group is ligated to Ca^2+^ (*12*). Despite the presence of excess IP_3_ in the crystallization buffer, IP_3_ binding to the nPH domain was not observed. This suggests that unlike PLCδ1, the nPH of PLCγ1 lacks a canonical PIP_2_ binding site. Similarly, Ca^2+^ binding to the C2 domain was not observed.

Although the overall fold is identical to the previously reported autoinhibited structure (PDB ID 6PBC), the PLCγ1-IP_3_ superimposes with an overall root mean square deviation of 0.716 Å highlighting the dynamic nature of this multidomain enzyme, particularly for the γSA. Furthermore, the new structure reveals detail for six newly resolved loops, resulting in the more complete structure (fig. S1). The two key regions of contact between the PLC core and the γSA, which stabilize autoinhibition, are outlined in fig. S1B. First, there are hydrophobic, electrostatic, and hydrogen bonded contacts between the sPH and the TIM barrel, centered on the hydrophobic ridge near the entrance to the active site. A second interface is formed between the C2 and cSH_2_ domains. M1166 and F1167, which form the hydrophobic tip of a loop on the C2 domain, bury into a hydrophobic pocket formed by F706, L746, and Y747 on the cSH2 domain. There is additionally a weak interaction between the EF-hand and nSH_2_ domains, stabilized by a single hydrogen bond between K175 and T576, which is positioned slightly differently from the previous structure (*18*).

### Autoinhibition prevents liposome binding

To investigate how autoinhibitory interactions may affect recruitment of PLCγ1 to membranes, we assessed the binding of either full-length PLCγ1 (PLCγ^FL^) or a core construct lacking the regulatory γSA (ΔSA or PLCγ^core^) to plasma membrane mimicking liposomes using a fluorescence resonance energy transfer (FRET)–based liposome binding assay. The PLCγ^core^ construct effectively bound to the liposomes under these conditions, but PLCγ^FL^ did not (Fig. 1B). To further investigate the binding of PLCγ^FL^ when autoinhibition is compromised by mutations, we used two variants (D1165H and S345F) previously characterized by an increase in basal PLC activity (*36*). In contrast to the wild-type (WT) PLCγ^FL^, both variants bound to the liposomes, and the binding of D1165H variant was comparable to that of PLCγ^core^ (Fig. 1B). Consistent with recent models, these data suggest a mechanism where autoinhibition obstructs membrane binding by the PLC core and support the idea that the PLCγ^core^ construct interacts with membranes in a similar way as PLCγ^FL^ when fully activated (*1*, *18*). These data are also consistent with observations that the PLCγ^core^ is both required and sufficient for maximal PLC activity (*37*–*39*). Consequently, the understanding of precise membrane interaction sites of the PLCγ^core^, compared to autoinhibitory interfaces, could reveal key properties of an active PLCγ at the membrane and provide further insights into the mechanism of activation.

### Characterizing the preferred membrane-bound orientation of the PLCγ1 core

**Fig. 2.**
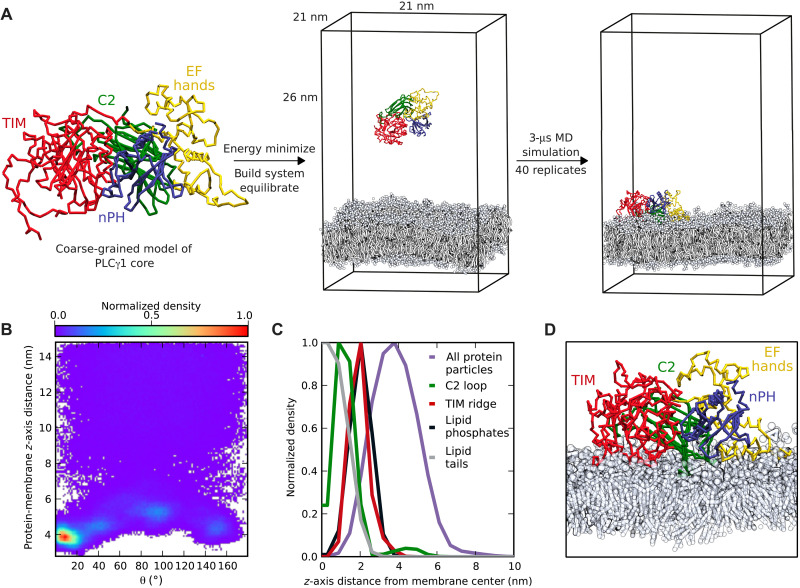
Characterization of membrane interaction of the PLCγ1 catalytic core using in silico techniques. (**A**) Schematic of the high-throughput CG MD simulation workflow used to study the membrane interactions of the PLC core. A CG model of PLCγ1^core^ was placed in a simulation box with explicit solvent, ions, and a symmetric model lipid bilayer (containing POPE, POPC, POPS, PIP_2_, PIP_3_, and cholesterol) and equilibrated. Starting with the same initial geometry but differing initial velocities, 40 replicate MD simulations of 3-μs duration were conducted using the Martini force field. Water molecules are omitted from the figure for clarity. (**B**) Two-dimensional histogram over all simulations, showing the density of states observed on a grid of protein-membrane distance and protein orientation (θ). (**C**) Distribution of protein groups along the *z* axis of the system in the preferred membrane-bound state. (**D**) Snapshot of the preferred membrane-bound state (at CG resolution), which positions the active site in contact with the membrane.

To characterize the membrane interaction of the PLCγ1 core, high-throughput coarse-grained (CG) MD simulations were conducted (Fig. 2A). A model of the PLCγ1 core domain was placed in a simulation box with a symmetric phosphoinositide-containing membrane model. The protein was placed above the membrane at a sufficient distance such that no forces from the membrane were experienced initially. Forty replicate simulations were generated with different initial velocities and run for 3 μs, allowing convergence in simulation data (fig. S2). Stable membrane binding was observed in all but one replicate, at a center of mass *z*-axis separation of the protein and the bilayer of about 4 to 5 nm (fig. S3).

The orientation of the PLCγ1 core throughout the simulation was quantified by measuring the angle (θ) between the *z* axis and a plane defined by the Cα particles of residues 416, 1011, and 1194 (fig. S4). Before encountering the membrane, the PLCγ1 core diffused freely in solution, suggesting that the initial geometry did not bias the subsequent membrane interaction (fig. S5). The selected backbone particles are in structured regions of the protein, and it is valid to assume minimal relative movement of these particles, due to the protein elastic restraint network used in the CG simulations. The plane was deliberately chosen to lie approximately parallel to the autoinhibitory interface observed in the PLCγ1^FL^ structure. Therefore, if the proposed mechanism of autoinhibition by obstruction of the membrane-binding interface of the core domains is correct, then a preferred membrane-bound orientation with small value of θ would be expected. Analysis of θ and the protein-membrane *z*-distance over all simulations frames reveals that the PLCγ1 core adopts a preferred membrane-bound orientation, with θ between 6° and 12° (Fig. 2B). Notably, this orientation positions the active site in productive contact with the membrane. Small populations of two alternative membrane-bound states were also observed. As these lowly populated alternative orientations position the active site away from the membrane, they are not likely to be physiologically relevant, although they may represent modes of interaction while PLCγ1 is bound to receptor tyrosine kinases (RTKs) and in proximity to the membrane.

Examining how the density of different particle groups was distributed along the *z* axis of the system in the preferred orientation (Fig. 2C), we found that the side chains of residues F344, L384, and L1018 of the TIM barrel hydrophobic ridge insert into the membrane interior, with the density penetrating into the region of the phospholipid tails. Membrane insertion of this ridge near the entrance of the active site has been previously suggested for PLCδ1, and the essential requirement for the PLCγ1 F344 has been demonstrated by the loss of PLC activity in the F334A variant (*12*, *40*, *41*). However, interactions with lipids at the ridge have not been defined at the molecular level. Furthermore, our analysis revealed membrane insertion of M1166 and F1167, located at the hydrophobic tip of a loop on the C2 domain, which is a previously undescribed feature of the PLCγ1 membrane interaction. Crucially, these two hydrophobic regions of the PLCγ core are involved in close contact with the γSA in the full-length autoinhibited structure. Previous studies of PLCγ1 disease-linked mutations, affecting M1166 and F1167 residues, suggest their potential impact on both processes (*18*).

Overall, the preferred membrane-binding interface (Fig. 2D) aligns with the autoinhibitory interface and is characterized by membrane insertion of the TIM barrel hydrophobic ridge and C2 hydrophobic loop. The nPH, EF hands, and part of the TIM barrel skim the surface of the membrane, and the enzyme is positioned productively, due to contact of the active site and hydrophobic ridge with the membrane.

### Identifying key sites for lipid interactions

**Fig. 3.**
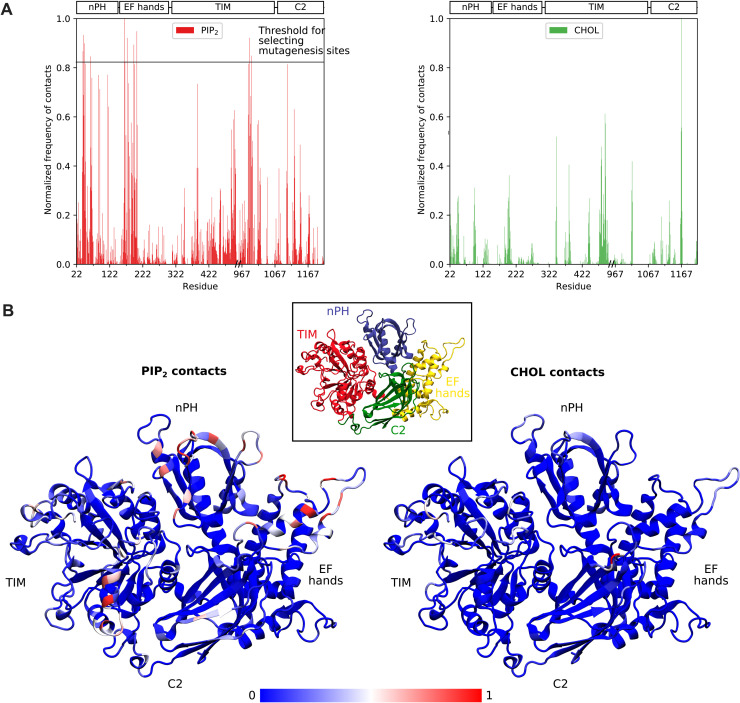
Key sites for interaction with PIP_2_ and cholesterol on PLCγ1^core^. (**A**) Normalized frequency of contacts with PIP_2_ headgroups (red) or cholesterol (green). Contacts were totalled across all simulation frames of the final 1 μs of simulation and normalized by dividing the total contacts at all residues by the total contacts for the residue with the most contacts. Residues with normalized PIP_2_ headgroup contacts above 0.8 were considered to be the most important residues for electrostatic interaction with anionic lipids and selected for mutation in subsequent experiments. (**B**) Data in (A) mapped onto the PLCγ1^core^ structure, where each residue is colored by its normalized frequency of contacts according to the blue-white-red scale shown. Inset: Structure of PLCγ1^core^ colored by domain.

Analysis of lipid contacts revealed hotspots for lipid interactions along the membrane-binding interface and an overall preference for interaction with phosphoinositides (fig. S6). In particular, we observed strong contacts with phosphoinositides on the nPH (β1-β2, β3-β4, and β5-β6 regions), EF hands (α1 and the surrounding loops), and helices (α5 and α5′) of the TIM barrel near the hydrophobic ridge (Fig. 3A). This result implies an unexpected mode of membrane interactions for the nPH and a role for the EF hands in membrane binding, not proposed before for PLCγ enzymes (*18*, *26*). The structural basis for these phosphoinositide interaction hotspots are clusters of cationic lysine and arginine residues that point toward the membrane surface and interact with anionic lipid headgroups.

In addition to electrostatic interactions with anionic lipids, we observed hotspots for contact with cholesterol on the C2 hydrophobic loop and the TIM barrel hydrophobic ridge (Fig. 3B), which reflects the insertion of these regions into the membrane as described above. Other domains, such as nPH and EF hands, show considerably less interaction with cholesterol. On the basis of these results, we propose a model of PLCγ1 core membrane binding, in which the C2 and TIM barrel domains undergo hydrophobic insertion into the membrane, while the nPH, EF hands, and a TIM barrel helix contribute to the membrane-bound state through electrostatic interactions with phosphoinositides and other anionic lipids at the membrane surface.

### Basic residues in the nPH, EF-hand, and TIM barrel domains are crucial for the affinity and orientation of the PLCγ1 core on the membrane

**Fig. 4.**
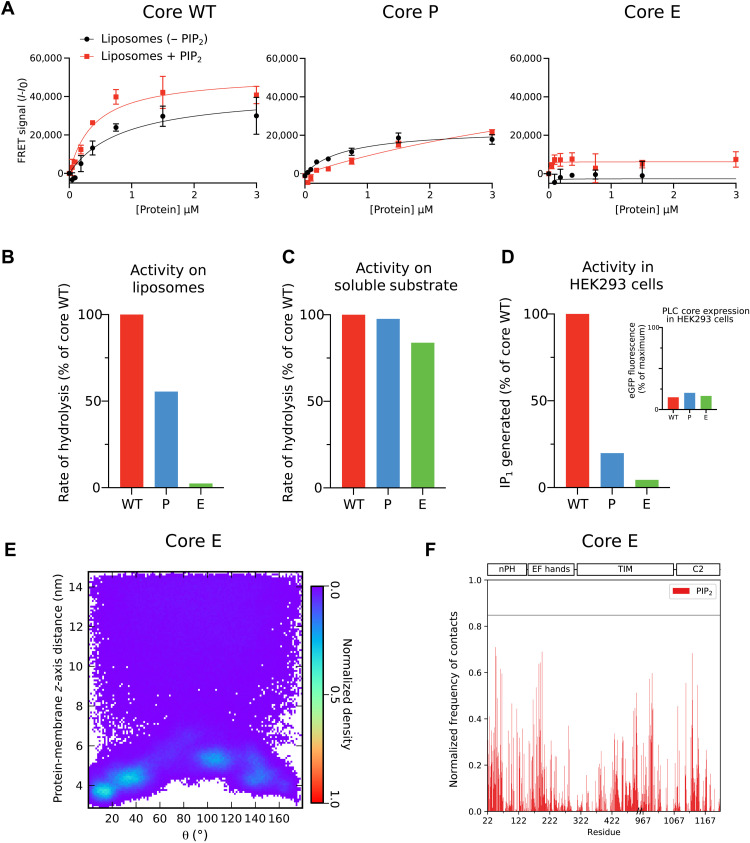
The identified lipid interaction sites are critical for the membrane binding and the activity of PLCγ1 on membrane-bound substrate. (**A**) FRET intensity between PLCγ1^core^ constructs and the fluorescent dansyl-phosphatidylserine (dPS) lipid probe embedded in PIP_2_-containing liposomes (red traces) or liposomes lacking PIP_2_ but which still contain anionic PS lipids (black traces). Data are shown for the PLCγ1 core WT and variants with alanine substitutions of arginine/lysine residues in the nPH (core P) and combine eight substitutions for the entire core (core E). (**B**) Relative rate of hydrolysis of liposome-embedded phosphatidylinositol (PI) by the PLCγ1^core^ constructs. The quantity of IP_1_ produced was measured at multiple time points using HTRF with the addition of a labeled IP_1_ probe and an anti-IP_1_–labeled cryptate antibody. Initial hydrolysis rates calculated by linear regression and normalized to the core WT. (**C**) Relative rate of hydrolysis of soluble Aldol 518 myo-inositol-1-phosphate (Aldol) by the PLCγ1^core^ constructs, monitored by measuring fluorescence of the reaction product. Equivalent enzyme concentrations that gave a linear activity were chosen, and the activity was presented as histograms for each variant normalized to the core WT. (**D**) Relative quantity of IP_1_ generated in human embryonic kidney (HEK) 293 cells lysed 49 hours after transfection with enhanced green fluorescent protein (eGFP)–tagged PLCγ1^core^ variants (core WT, core P, and core E). IP_1_ produced was quantified using the HTRF assay previously described. Inset: Core variant expression levels presented as histograms showing the eGFP fluorescence normalized to the maximum eGFP expressed in a control well in the experiment. (**E**) Density of protein-membrane distance and orientation (θ) states observed in simulations of a PLCγ1^core^ variant, core E. (**F**) Normalized frequency of contacts of core E with PIP_2_ headgroups during simulation; the PIP_2_ contacts are substantially reduced relative to core WT, as indicated by the original threshold line.

The clusters of basic residues that point toward the membrane surface, involved in electrostatic interactions, could be critical for the overall membrane binding. Therefore, we extended our analyses, including different experimental approaches, to investigate this new hypothesis. On the basis of contacts observed during simulations, we identified the most important residues for interaction with anionic lipids on the nPH (K41, K42, and R45), EF hands (R166, K175, R195, and R204), and TIM barrel (K990) domains. To test whether these residues are important for membrane binding and productive catalytic activity of PLCγ1, we conducted further in vitro, in cell, and in silico studies using a panel of PLCγ1 core constructs that contain lysine/arginine to alanine substitutions at the identified residues. An initial finding related to phosphoinositide/anionic lipid headgroup affinity, based on Heparin Sepharose mobility, demonstrated decreasing affinity for negatively charged heparin with each additional mutation (fig. S7). We focused on three variants: (i) the core with the WT residues, designated as core WT; (ii) the core with the three substitutions in the nPH domain, designated as core P; and (iii) the core with all eight substitutions, designated as core E. FRET liposome binding assays [performed with catalytically inactive versions (H335A)] demonstrated that the core WT construct has a preference for PIP_2_-containing liposomes over liposomes containing anionic PS but not phosphoinositides (Fig. 4A). The core P mutant has reduced affinity for liposomes. This demonstrates the importance of the identified nPH residues (which lie outside the canonical PIP binding pocket for PH domains) for membrane affinity and phosphoinositide targeting. The core E variant displayed minimal binding to liposomes without PIP_2_ and greatly reduced binding to PIP_2_-containing liposomes compared to the core WT. The same variants of the PLCγ1 core were also assessed for their PLC activity using different assay systems (Fig. 4, B to D, and fig. S8). When presented with liposome-bound substrate, the PLC activity was reduced by almost 50% in core P and by more than 90% for core E, relative to core WT (Fig. 4B and fig. S8A). In contrast, the activity toward soluble substrate was retained for both mutants (Fig. 4C and fig. S8B), indicating that the loss of activity is caused by reduction in membrane affinity and not loss of integrity of the active site. Furthermore, these findings are supported by reduction of activity of core P and core E observed in the cellular context (Fig. 4D and fig. S8C). These experimental data are consistent with further MD simulations of the mutants. Compared to core WT, simulations conducted using a core E model showed a depopulation of the preferred membrane-bound orientation (Fig. 4E) and a reduction in lipid contacts (Fig. 4F)—consequences of reduced membrane affinity and diminished ability to maintain a catalytically productive orientation without the crucial electrostatic interactions with anionic lipids.

### An atomistic model of the PLCγ1 core on the membrane

**Fig. 5.**
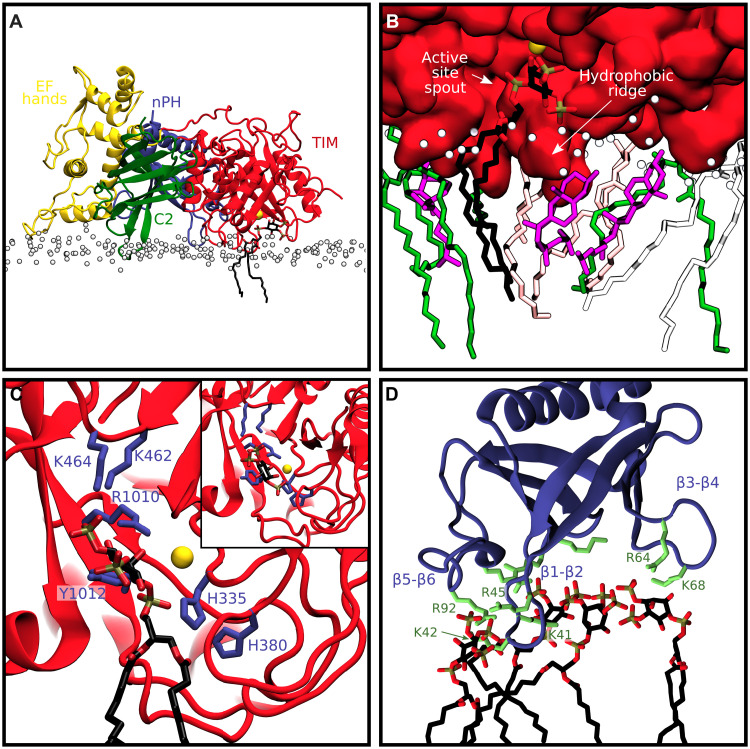
A model of PLCγ1^core^ at the membrane based on atomistic MD simulations. (**A**) Simulation snapshot of the PLCγ1^core^ domains (nPH, blue; EF hands, yellow; TIM, red; C2, green) bound to the membrane with PIP_2_ (stick representation) and Ca^2+^ (yellow sphere) at the active site. P1 lipid phosphate particles are shown as white spheres. (**B**) Overview of the active site (TIM barrel domain shown as red surface representation) with bound PIP_2_. The hydrophobic ridge interacts with the tails of a number of additional lipid molecules (cholesterol, purple; POPE, green; POPC, salmon; POPS, white). (**C**) Zoom on the active site in the simulation snapshot, with notable residues (blue sticks) interacting with the PIP_2_ headgroup. Inset shows the crystal structure of PLCγ1 with IP_3_ in the active site (see fig. S10A for detail). (**D**) View of the nPH domain interacting with multiple phosphoinositides at noncanonical sites during the simulation. Lysine and arginine residues are shown as green sticks.

An atomistic model of the PLCγ1 core on the membrane, with PIP_2_ at the active site, was obtained by backmapping of the bound CG system to an all-atom representation for the CHARMM36 force field (fig. S9). The membrane-bound system was simulated for 1 μs of atomistic simulation, during which time the PLCγ1 remained bound to the membrane in the identified preferred orientation. From this simulation, we present the first atomistic model of the PLC core at the membrane surface, bound to its PIP_2_ substrate (Fig. 5A). Examination of the active site (Fig. 5B) revealed a solvent-accessible cavity that accommodates the PIP_2_ headgroup, while the lipid acyl tails slot into a groove formed by the hydrophobic ridge. The hydrophobic ridge is inserted into the membrane and has additional interactions with cholesterol and the acyl tails of other phospholipid molecules. The dual nature of the active site—consisting of the solvent-accessible spout and hydrophobic ridge—allows accommodation of the amphiphilic lipid substrate and likely presents two separate exit pathways for the inositol phosphate (hydrophilic) and diacylglycerol (hydrophobic) reaction products, as has previously been suggested (*12*).

Details of interaction between the PIP_2_ headgroup and the active site residues, shown in Fig. 5C and aligned in fig. S10A, are consistent with those seen for the IP_3_ in the crystal structure (Fig. 5C, inset, and fig. S10A). The PIP_2_ headgroup observed in the simulations, however, is not as deep in the active site as observed for IP_3_ in the structure. To date, no structure of a PLC has been solved in complex with a complete lipid (only the inositol phosphate), and our model provides such a structure. It is possible that the interactions of the lipid tails with the hydrophobic ridge result in this small difference by preventing deeper penetration of the headgroup. On the other hand, our model of the active site geometry may not be fully optimized because of force field limitations and difficulty sampling such a large and computationally demanding simulation system. To overcome this limitation, further restrained MD simulations were conducted, with four distance restraints (see Materials and Methods) applied between the PIP_2_ headgroup and the protein based on the geometry of the inositol group observed in the structure. During the restrained simulations, the PIP_2_ headgroup quickly moved deeper into the active site to match the geometry of inositol observed in the crystal structure (fig. S10B). The lipid acyl tails remained in contact with the hydrophobic ridge and did not perturb the ridge or the rest of the active site.

To obtain the atomistic model, it was important not to include the two linkers between the TIM barrel and the sPH domain (residues 471 to 488 and 934 to 947) during CG simulations, as we observed a tendency of these loops to move toward the active site and occlude entry of PIP_2_ (fig. S9). To see whether the linker region prevents substrate access to the active site, the end frame of one of the simulations including the loops was selected (due to the presence of a PIP_2_ molecule proximal to the active site in the preferred orientation), the linker loop residues were removed, and the CG simulation was extended for a further 1 μs. During this simulation, PIP_2_ entered and remained stably bound in the active site (fig. S9); this was used as the starting point for backmapping to the atomistic representation described above. The XY-linker loop of the PLCβ family is known to inhibit substrate access to the active site in the inactive form of PLCβ, and this may also be the case for the TIM barrel/sPH loops in the PLCγ family. This predicted occlusion occurs because of the specific loop positioning in the context of an autoinhibited enzyme, while the PLCγ^core^ containing these loops retains high, constitutive PLC activity (*38*, *39*). Alternatively, the observed active site occlusion could be an artifact of the CG simulation method, which has limited accuracy in modeling conformational dynamics, and a tendency to overestimate protein-protein interactions (*42*).

In addition to insights into the TIM barrel active site binding to PIP_2_, we have also obtained further detail for the C2 domain insertion, centered on M1166 and F1167. Structural and sequence alignment of the PLCγ1 C2 domain and PLCδ1 C2 domain (fig. S11A) suggests that the PLCγ1 C2 domain may have lost Ca^2+^ binding functionality, and no Ca^2+^ binding was observed in the crystal structure, at this region, despite excess Ca^2+^. D653 in PLCδ1 has previously shown to be essential for Ca^2+^-binding, yet in PLCγ1, this position is replaced by a cysteine (fig. S11A) (*43*, *44*). Furthermore, comparable residues to the membrane-inserting M1166 and F1167 of PLCγ1 are not found in the PLCδ1 loop regions. Together, these analyses suggest divergence in the mode of membrane interaction of the C2 domains in these two families. Further detail for the EF hands, highlighting interactions with anionic lipids, has also been obtained (fig. S11B). Involvement of the EF hands in membrane interactions, including binding to anionic lipids and PIP_2_ mediated by arginine and lysine residues, has been suggested for PLCδ1 and PLCζ1; however, these protein/lipid interactions have not been precisely mapped (*45*, *46*).

### The nPH interacts with anionic lipids at multiple noncanonical sites

During simulations, we observed the nPH interacting with multiple anionic lipids at noncanonical sites (Fig. 5D). These interactions are facilitated by clusters of basic residues that point toward the membrane surface and engage in hydrogen bonding and electrostatic interactions with anionic lipid headgroups. K42, K45, and R92 comprise one binding site between the β1-β2 and β5-β6 loops. A second binding site is formed by R64 and K68, and a further region, which accommodates multiple lipid headgroups, is formed by K41, R88, R117, and K118 toward the back of the PH domain as shown in Fig. 5D. Two disease-linked mutations (E47K and R48W) in the nPH are outside these regions and have very little or no effect on the PLCγ1 activity (*18*, *36*, *47*).

Structural alignment and comparison with previous simulations of the PLCδ1 and ArhGAP9 PH domains (fig. S12) suggest that PLCγ1 lacks the lysine and arginine residues present in the canonical pocket of PLCδ1 PH and which appear to be necessary for strong and specific PIP_2_ binding and in some other PH domains for the binding of PIP_3_ (*34*). The PLCγ1 nPH is more akin to the ArhGAP9 PH domain, which points clusters of basic residues outward toward the membrane, and has a noncanonical PIP binding pocket between the β1-β2 and β5-β6 loops (*48*). This analysis reconciles our findings that the nPH is critical for liposome binding and catalytic activity (Fig. 4, A to D) and yet has no IP_3_ binding site detected in the crystal structure. Rather than specific strong binding to PIP_2_, we propose that it contributes to membrane association through electrostatic interaction with multiple anionic lipids. In contrast to previous observations (*26*), a selective binding of PIP_3_ to a canonical binding pocket is also unlikely.

### Identifying possible interfaces of γSA-membrane interaction

The positioning of the γSA domains in the active form of PLCγ1 is unknown, and it is possible that at least some of the γSA could contribute toward membrane interaction. To identify the likely interfaces of such potential membrane interactions, further CG membrane-binding simulations were conducted for the structures of the sPH domain, tandem nSH2-cSH2, and the SH3 domain. We note that these simulations do not provide a measure of the strength of the membrane interaction but rather identify the most plausible mode of membrane interaction should it occur. For each structure, 20 replicate simulations of 1 μs were conducted. As an indicator of the membrane-binding interface, we examined the normalized frequency of contacts with POPE headgroups for each structure (fig. S13). The sPH domain interacted with the membrane primarily through its C-terminal α-helix, which points a cluster of basic residues toward the membrane surface; PH domains typically interact at the opposite, open face of the β-barrel. This interaction is physiologically plausible as the sPH is likely to be positioned close to the membrane in the active form. For the cSH_2_ domain, membrane interactions were localized around its phosphotyrosine binding site. In the active form, this region would be bound to the segment of cSH2/SH3 linker surrounding pY783; therefore, it is not likely to be a physiological site of membrane interaction (*39*, *49*). In contrast with the PLC core, overall, the γSA domains do not use the same interface for autoinhibition and membrane interaction.

## DISCUSSION

**Fig. 6.**
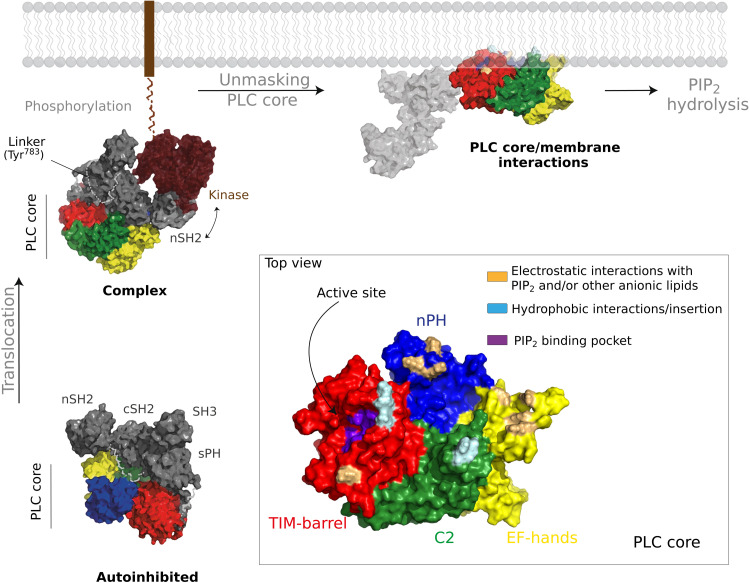
Model for activation and membrane interactions of PLCγ1. The depiction of the autoinhibited PLCγ1 is based on crystal structures (PDB IDs: 6PBC and 7Z3J) (bottom left); it involves interactions between the regulatory region (γSA) and the PLC core. In the best-defined activation route by RTKs, the translocation of PLCγ1 to the membrane proximity is mediated by the recruitment to activated RTKs (e.g., FGFR1). The depiction of the PLCγ1/FGFR1 complex is based on structural insights (EMD-10288 and PDB ID: 3GQI) (top left). The nSH2 domain in the γSA binds to pTyr766 in the C-terminal tail of FGFR1. It is envisaged that subsequent destabilization of γSA/PLC core interactions facilitates phosphorylation of Tyr^783^ in the cSH2/SH3 linker. Binding of pTyr^783^ to the cSH2 disrupts the cSH2/C2 autoinhibitory interface providing a key trigger for a large-scale rearrangement of the γSA with respect to the PLC core. On the basis of our data, a complete “unmasking” of the PLC core surface is required for a productive orientation of the PLC core and the hydrolysis of membrane-resident PIP_2_. In the depiction of PLCγ1 at the membrane (top right), the γSA position and shape are arbitrary (indicated by the light gray color). For the PLC core, the amino acid residues involved in interactions between the PLC core and the membrane, revealed here, are highlighted (inset).

Insights into the mechanisms by which PLCγ enzymes are activated and recruited to the plasma membrane are critical for understanding their roles in physiology and disease as well as for therapeutic intervention. By combining MD simulations with experimental approaches, we here elucidate key properties of membrane interactions by the PLCγ1 catalytic core domains, resulting in productive substrate hydrolysis by an active enzyme. Our findings are summarized in the context of an overview for PLCγ activation (Fig. 6).

Among PLCs, PLCγ1 and PLCγ2 are uniquely characterized by a large regulatory array (γSA) inserted between the X and Y boxes of the TIM barrel domain, and they are the only PLCs known to be activated via tyrosine phosphorylation (*1*). Furthermore, compared to other PLC families, the interactions between the regulatory domains and the PLC core are quite extensive resulting in strong autoinhibition. Although the PLC core is shared with other PLC families and has similar overall architecture, domains in the PLC core can have specific binding properties for various regulatory proteins or other ligands, distinct from those commonly recognized by these modular domains in other proteins. For example, in contrast to PIP_2_ binding by the PLCδ1 PH domain, the PH domain from PLCβ2 mediates binding of the small guanosine triphosphatase Rac (*13*). Similarly, Ca^2+^ binding by the EF hands has not been generally documented for PLC enzymes; instead, the EF hands in PLCβ enzymes underpin the function that accelerates guanosine triphosphate hydrolysis by Gαq, and in PLCδ1 and PLCζ1, facilitate membrane interactions (*45*, *46*, *50*). Therefore, it is important to determine specific functional properties for the core domains in each PLC family. Overall, our data reveal such distinct functional properties of PLCγ1 core domains with respect to membrane interactions. We show that multiple defined points of membrane interaction act in synergy, incorporating both hydrophobic and electrostatic components, and result in a productive orientation of the core on the membrane, with the PIP_2_ substrate bound to the active site. (Figs. 3 to 5). Consistent with previous data for other PLC families (*12*), we found that the hydrophobic ridge of the TIM barrel inserts into the membrane and, in fact, forms a channel that accommodates the lipid tail (Fig. 5). Furthermore, we identified a previously unknown membrane-inserting loop on the C2 domain (Figs. 2C and 3 and fig. S11). With respect to interaction with inositol lipids and other anionic lipids, we show that the nPH helps to drive membrane association not via a canonical binding pocket for inositol lipids, as found for the PLCδ1 PH domain, but through electrostatic interaction with multiple anionic lipid headgroups at noncanonical sites (Figs. 4 and 5 and fig. S12). Last, we found that the EF hands also drive membrane association through similar interactions with anionic lipids (Fig. 3 and fig. S11); no role of the EF hands in PLCγ has been previously identified. These membrane-targeting functions of the nPH and EF hands are crucial for the membrane binding and activity of PLCγ1. The membrane interactions of the PLC core are summarized in Fig. 6 (inset).

Considering the unique autoinhibitory regions on the core of PLCγ1, it is interesting that membrane-interacting surfaces, revealed in detail in this study, largely overlap with autoinhibitory interfaces (fig. S14). Key residues (K175, F344, L384, L1018, M1166, and F1167) clustered in areas involved in PLC core/γSA contacts in the autoinhibited structures were found to be important sites of membrane interactions also. Our data, therefore, strongly support a mechanism of PLCγ1 autoinhibition via an extensive obstruction of the membrane-binding surfaces on the PLC core. A substantial change upon activation must completely unmask the core surfaces, to produce a conformation that is competent for membrane binding. In addition to physiological activation, some of the strongly activating mutations could have a similar impact; we observed comparable liposome binding for PLCγ1^core^ and PLCγ1^FL (D1165H)^ variants (Fig. 1B), which supports the idea that activation exposes much of the inhibited core interface.

The overlap of the autoinhibitory and membrane-interacting interfaces in PLCγ extends the current framework for the mechanistic interpretation of the impact of various disease-linked mutations. For example, the activating S345F hotspot mutation in T cell lymphoma has been implicated in disruption of autoinhibitory sPH/TIM barrel interactions (*18*, *19*). However, this mutation will also increase the hydrophobicity of the TIM barrel hydrophobic ridge and the loop regions that, based on our data (Fig. 5), interact with the acyl tails of PIP_2_ bound at the active site. Similarly, considering this overlap, a rational design of potential inhibitors selective for PLCγ enzymes, by targeting unique features involved in membrane binding rather than the conserved active site, may result in disruption of the autoinhibitory regions and overall enzyme activation.

On the basis of the current evidence, the release of autoinhibitory interactions in PLCγ enzymes is mediated by protein-protein interactions and phosphorylation of the key tyrosine residue (Y783 in PLCγ1) (*1*). In the PLCβ family, an additional role of the membrane interface in activation has been extensively studied (*16*, *21*, *22*, *51*, *52*). Specifically, a large cluster of acidic residues in the XY-linker occludes the active site and could be displaced in the proximity of the negatively charged membrane to enable access to PIP_2_ substrate. The XY-linker from PLCβ2 can also inhibit the PLCγ^core^ (*38*). Similar occlusion of the PLCγ1 active site in the autoinhibited enzyme could involve the TIM barrel/sPH linkers (fig. S9); however, these linkers contain few negatively charged amino acid residues, and the repositioning of these linkers during activation is likely to be mechanistically different (*38*, *39*). Although interfacial activation could play a role in regulation of PLCγ enzymes, this possibility and the precise mechanism remain unclear.

An overall activation mechanism for PLCγ enzymes presented in Fig. 6 mainly considers activation by RTKs and particularly the evidence obtained for the fibroblast growth factor receptor 1 (FGFR1)/PLCγ1 interaction. Recently, studies of PLCγ activation in the context of signaling in immune cell types supported models with additional complexity related to different PLCγ conformations and interactions with adapter proteins, non-RTKs, and membrane mimetics (*53*–*55*). However, some of the common aspects of PLCγ membrane interactions remain unresolved. Notably, it is not clear whether the membrane-bound state, involving the PLC core/membrane interactions, may be further stabilized by direct interactions of the γSA domains with lipids (suggested by our simulations; fig. S13) or by indirect interactions with membrane-bound RTKs and adapters. With respect to indirect interactions, our previous work has suggested that the active conformation of PLCγ1 may be released from FGFR1 following phosphorylation and independently interact with the membrane (*39*). Nevertheless, the data obtained in this study reveal the contacts on the PLC core that are required for productive membrane orientation, regardless of other interactions by the entire PLCγ1 protein. They also imply extensive rearrangements needed to unmask the PLC core surfaces that are involved in both complex autoinhibitory and membrane interactions. The insights gained here further support the capability of MD simulations to provide realistic and valuable insight into membrane biology (*28*, *56*).

## MATERIALS AND METHODS

### Constructs, protein expression, and purification

Full-length human PLCγ1 and its S345F and D1165H variants, cloned in the pTriEx4(Gateway) vector, have been described previously (*19*). Deletion variant lacking the γSA regulatory region (amino acids 488 to 933), also referred to as PLC core, was generated using polymerase chain reaction as described previously (*39*). For constructs where PLCγ1 was fused to enhanced green fluorescent protein (eGFP), an open reading frame (ORF) encoding eGFP was inserted N-terminally to the PLCγ1 using ligation-independent cloning and verified by sequencing. All amino acid substitutions were generated using a site-directed mutagenesis kit (Agilent), and the ORFs of all clones were fully sequenced. The construct for protein crystallization of rat PLCγ1 (98% amino acid identity with human PLCγ1) included amino acids 21 to 765 and 791 to 1215, joined by a SGS linker, as originally described (*18*). The construct contains a TeV protease recognition sequence that after cleavage leaves two nonnative amino acid residues at the N terminus (serine and glutamine).

All PLCγ1 proteins were expressed in *Escherichia coli* strain C41 (DE3). Cells were transformed, and colonies were grown up in 2xYT to an optical density at 600 nm of 0.5 at 37°C. Cultures were cooled for 2 hours at 15°C, and expression was induced by the addition of 100 mM isopropyl-β-d-thiogalactopyranoside and left overnight. Cells were harvested by centrifugation, and pellets were stored at −20°C until use.

Cell pellets from 1 liter of culture were lysed by the addition of 25 ml of lysis buffer [25 mM tris-Cl, 250 mM NaCl, 40 mM imidazole, 10 mM benzamidine, 1 mM MgCl_2_, and 100 ml of CaCl_2_ (pH 8.0)] containing egg white lysozyme (0.25 mg/ml), with constant agitation at 4°C. After 30 min, 5 ml of 10% (v/v) Triton X-100 was added, and lysis was continued for another 30 min. Cell lysates were centrifuged for 1 hour at 20,000 rpm in a Beckman JA-25.50 rotor at 4°C.

#### Purification of crystallizable rat PLCγ1

Clarified lysate was applied to a 5-ml HisTrap column on an Akta Purifier (Cytiva) system equilibrated in His buffer A [25 mM tris-Cl, 500 mM NaCl, 40 mM imidazole, and 1 mM TCEP (pH 8.0)] and washed for 20 column volumes in His buffer A to remove contaminants. Bound proteins were eluted with the addition of His buffer B [25 mM tris-Cl, 500 mM NaCl, 500 mM imidazole, and 1 mM TCEP (pH 8.0)]. The concentration of eluted protein was determined using Bradford reagent, and TeV protease was added giving a final ratio of TeV to protein of 1:50. The protein was dialyzed overnight at 4°C against 1 liter of dialysis buffer [25 mM tris-Cl, 20 mM NaCl, 10% (v/v) glycerol, 1 mM TCEP, 1 mM EDTA, and 0.1 mM EGTA (pH 8.0)]. The following day, the protein was applied to a 5-ml HiTrap Heparin column (Cytiva) equilibrated in heparin buffer A [25 mM tris-Cl, 20 mM NaCl, and 1 mM TCEP (pH 8.0)] and washed with 4 column volumes of the same buffer. Proteins were eluted with a gradient over 20 column volumes with heparin buffer B [25 mM tris-Cl, 500 mM NaCl, and 1 mM TCEP (pH 8.0)]. Eluted PLCγ1 was then injected onto a Superdex 200 preparative column (Cytiva) equilibrated in crystal gel filtration buffer [20 mM Hepes-NaOH, 150 mM NaCl, and 2 mM dithiothreitol (pH 7.5)], and 1CV of an isocratic gradient resolved a peak of purified PLCγ1. Proteins were concentrated to around 40 mg/ml using an Amicon Ultra-15 centrifugal filter unit (Merck) following the manufacturer’s instructions, aliquoted, snap-frozen in liquid nitrogen, and stored at −80°C until use.

#### Purification of all other variants

Clarified lysate was applied to a 5-ml HisTrap column on an Akta Purifier (Cytiva) system equilibrated in His buffer A [25 mM tris-Cl, 500 mM NaCl, 40 mM imidazole, and 1 mM TCEP (pH 8.0)] and washed for 20 column volumes in His buffer A to remove contaminants. Bound proteins were eluted with the addition of His buffer B [25 mM tris-Cl, 500 mM NaCl, 500 mM imidazole, and 1 mM TCEP (pH 8.0)] and then diluted fourfold with dilution buffer [25 mM tris-Cl and 1 mM TCEP (pH 8.0)]. Diluted proteins were then applied to a 5-ml HiTrap Heparin column (Cytiva) equilibrated in heparin buffer A [25 mM tris-Cl, 20 mM NaCl, and 1 mM TCEP (pH 8.0)] and washed with 4 column volumes of the same buffer. Proteins were eluted with a gradient over 20 column volumes with heparin buffer B [25 mM tris-Cl, 500 mM NaCl, and 1 mM TCEP (pH 8.0)]. Eluted PLCγ1 was then injected onto a Superdex 200 preparative column (Cytiva) equilibrated in gel filtration buffer [25 mM Hepes-NaOH, 150 mM NaCl, 5% (v/v) glycerol, and 2 mM TCEP (pH 7.5)], and 1CV of an isocratic gradient resolved a peak of purified PLCγ1. Proteins were generally concentrated to around 5 mg/ml using an Amicon Ultra-15 centrifugal filter unit (Merck) following the manufacturer’s instructions, aliquoted, snap-frozen in liquid nitrogen, and stored at −80°C until use.

### Protein crystallization

The protein for crystallization was prepared from a frozen stock (41 mg/ml). The protein was diluted to a final concentration of 7.0 mg/ml in the crystal construct gel filtration buffer, containing, in addition, 2 mM IP_3_ and 2 mM CaCl_2_. The crystallization experiment was set up immediately using a 24-well VDX crystallization plate (Hampton Research) and the hanging-drop vapor-diffusion technique. Equal volumes (1 μl) of protein and reservoir solution were mixed against a 500-μl reservoir solution at 20°C. A sparse matrix layout was designed for the crystallization trials by varying the concentrations of polyethylene glycol 3350 (PEG 3350) from 17 to 22% (w/v) and the pH of 0.1 M citric acid bis-tris propane (CBTP) buffer from 6.8 to 7.5. The best diffracting crystal for the complex was found in a condition containing 18.7% PEG 3350 and 0.1 M CBTP (pH 7.0). Before data collection, harvested crystals were immersed in a solution containing the precipitant mixture and 12% (v/v) 2-methyl-2,4-pentanediol and cryo-cooled in liquid nitrogen.

### Crystallographic data collection and refinement

A crystal of the rPLCγ1/IP_3_ complex was measured at the ID23-1 beamline (ESRF, Grenoble, France) at 100 K and processed by the XDS package (*57*). The crystal belonged to the *P 2_1_ 2_1_ 2_1_* space group with a solvent content of 52.9%, corresponding to one protein molecule in the asymmetric unit. The structure was determined by molecular replacement using MOLREP (*58*) using the previously determined structure of rPLCγ1 with PDB ID 6PBC as a search model.

Initial rigid body refinement of the molecular replacement model using the phenix.refine program of the PHENIX suite (*59*) revealed extra density near the active site that was modeled as IP_3_. Further iteration of TLS/maximum-likelihood refinement and manual model building and inspection using COOT (*60*), the model converged to a final *R*_work_/*R*_free_ of 0.2241/0.2551 at a maximum resolution of 2.00 Å. Data collection and refinement statistics are summarized in table S1.

### Liposome preparation

Liposomes were prepared and stored at a final concentration of 2 mg/ml. The following lipid components in organic solvent were mixed in the following ratios for different liposomes:

1) Control FRET liposomes: 20% brain phosphatidylserine (PS), 40% brain phosphatidylethanolamine (PE), 15% brain phosphatidylcholine (PC), 10% cholesterol, 5% sphingomyelin, and 10% dansyl-phosphatidylserine (dPS) (w/v) (Avanti Polar Lipids).

2) PIP_2_ FRET liposomes: 20% PS, 35% PE, 15% PC, 10% cholesterol, 5% sphingomyelin, 5% phosphatidylinositol (4,5) bisphosphate [PI(4,5)P_2_], and 10% dPS.

3) PLCγ activity assay liposomes: 20% PS, 45% PE, 15% PC, 10% cholesterol, 5% sphingomyelin, and 5% phosphatidylinositol (PI).

The organic solvent was removed from the lipids in glass test tubes using a stream of nitrogen gas. The remaining solvent was removed by drying the lipids under vacuum for 2 hours at room temperature. Lipids were resuspended in liposome buffer [20 mM Hepes-KOH, 100 mM KCl, and 1 mM EGTA (pH 7.5)] by vortexing and bath sonication and then transferred to a microfuge tube. The liposomes were subjected to 11 freeze-thaw cycles between liquid nitrogen and a water bath at 42°C. Last, the liposomes were extruded through a 100-nm filter, and this process was repeated 10 more times. Liposomes were snap-frozen in liquid nitrogen and stored at −80°C.

### Protein-liposome FRET assay

Liposome binding was monitored by observing the FRET interaction between an 18:1

dPS fluorescent lipid probe and intrinsic tryptophan fluorescence of the PLCγ1 variants. Liposomes were defrosted and kept at room temperature until use. Assay components were diluted in FRET buffer [30 mM Hepes-NaOH and 50 mM NaCl (pH 7.5)]. Liposomes were diluted to 100 mg/ml. Proteins were diluted to a maximum concentration of 6 mM, and then a further sixfold dilution series was prepared. In a black, low binding 384-well plate (Greiner Bio-One), 5 ml of protein (or buffer only for *I*_0_ measurements) and 5 ml of liposomes were mixed in triplicate. Plates were sealed and then incubated for 1 hour at room temperature. FRET was measured on a PHERAstar plate reader (BMG Labtech) using a 280-nm excitation filter and 350/520-nm emission filters to measure Trp and dPS emissions, respectively. The FRET signal shown in figures is (*I*-*I*_0_), where *I* is the intensity at 520 nm, and where *I*_0_ is the intensity for a solution with lipid only (without protein). Binding curves were fit with a one site-specific binding curve using GraphPad Prism (Prism v9.3.0 for Mac). Association constants (*K*_a_) have not been reported since PLCγ^FL-WT^ did not display any measurable binding. Each analysis was repeated twice (biological replicates) with three replicates in each experiment (technical replicates).

### Measurement of PLC activity in vitro

Determination of PLC activity in vitro was performed by two methods. First, the PLC-driven hydrolysis of PI incorporated in liposomes was monitored by quantifying the production of inositol phosphate (IP_1_) using the IPone kit (CisBio) in an end point assay format. Second, the real-time hydrolysis of the synthetic substrate Aldol 518 myo-inositol-1-phosphate (abbreviated to Aldol) (Biosynth) was monitored in a continuous assay format. Essentially, both methods used the following assay buffer [20 mM Hepes-KOH, 70 mM KCl, 3 mM EGTA, 2.97 mM CaCl_2_, 2 mM TCEP, and fatty acid–free bovine serum albumin (BSA; 50 μg/ml) (pH 7.0)].

For the measurement of IP_1_ production, liposomes containing PI as outlined above were thawed, diluted to lipid (0.2 mg/ml) in assay buffer, and stored at room temperature. The PLC proteins were assayed at a final concentration of 50 nM in a total assay volume of 150 μl. Samples of 15 μl were removed at various time intervals, and the reaction was stopped by the addition of 20 μl of stop buffer [50 mM EGTA and 0.6% (w/v) sodium cholate (pH 8.0)] and heat denaturation at 80°C for 2 min. The amount of IP_1_ was quantified through the addition of a labeled IP_1_ probe and an anti-IP_1_–labeled cryptate antibody and monitored by Homogeneous Time Resolved Fluorescence (HTRF) as outlined in the manufacturer’s instructions. The amount of IP_1_ produced was calculated by interpolation from a standard curve. The initial rate of hydrolysis was calculated using linear regression and plotted as histograms for each variant normalized to the core^WT^ variant activity. Each analysis was repeated twice (biological replicates) with three replicates in each experiment (technical replicates).

In the continuous assay format, the enzymatic activity of core variants was quantified using the soluble Aldol substrate. Activity was measured in low-volume black 384-well plates (Greiner Bio-One). A final assay volume of 20 μl contained 25 μM Aldol substrate in assay buffer, 125 μM Aldol 355 fluorescence enhancer (Biosynth), and a serial dilution of PLC core variants. All were performed in triplicate. Production of fluorescent product was monitored in a CLARIOstar multimode plate reader (BMG Labtech) with an excitation filter of 510 nm and an emission filter of 618 nm. Equivalent enzyme concentrations that gave a linear activity were chosen, and the activity was presented as histograms for each variant normalized to the core^WT^ variant activity. Each analysis was repeated twice (biological replicates) with three replicates in each experiment (technical replicates).

### Measurement of PLC activity in cells

For the measurements of PLC activity, human embryonic kidney (HEK) 293 cells were cultured in Dulbecco’s modified Eagle’s medium (Sigma-Aldrich) containing 10% (v/v) fetal bovine serum (FBS) and 2.5 mM glutamine (growth media). Cells were grown as a monolayer at 37°C in 5% CO_2_. HEK293 cells were seeded into 96-well plates at a density of 7500 cells per well in 0.1 ml of growth media and incubated overnight. Fresh medium was applied, and the cells were transfected with plasmid DNA at 50 ng per well, which had been diluted in 5 μl of jetPRIME buffer and 0.2 μl of jetPRIME (Polyplus) that were prepared as instructed by the manufacturer. The DNA concentration was kept constant by adding empty plasmid. Each PLCγ1^core^ construct was transfected at several concentrations in triplicate. Twenty-four hours after transfection, the media was removed and replaced with growth media without FBS but containing 0.25% (w/v) fatty acid–free BSA. The HEK293 cells were then incubated for a further 24 hours. Subsequently, the media was replaced with growth media without FBS but containing 50 mM LiCl and incubated for a further 1 hour. The media was aspirated and replaced by 25 μl of 2× stimulation buffer (CisBio) followed by 25 μl of lysis buffer (CisBio). The cells were lysed for 10 min at room temperature on an orbital shaker. Fourteen microliters of the cell lysate was pipetted into white 384-well plates (Greiner Bio-One) followed by 3 μl of IP1-d2 and 3 μl of anti-IP_1_-cryptate (Cisbio). The plate was sealed and incubated at room temperature for 1 hour. The plate was read on a PHERAstar (BMG Labtech) plate reader in HTRF mode using a 337-nm excitation filter and 620/665-nm emission filters. The data were converted to IP_1_ concentration using a standard curve generated following the manufacturer’s instructions.

Expressed PLCγ1^core^ constructs were eGFP tagged. Therefore, the same plate analyzed for IP_1_ concentration was reread for recombinant protein expression using a CLARIOstar plate reader (BMG Labtech) with 470-nm excitation and 515-nm emission. PLC activity data are presented as histograms showing the IP_1_ generated normalized to the value for the core WT variant. Core variant expression levels are also presented as histograms showing the eGFP fluorescence normalized to the maximum eGFP expressed in a well in that experiment. Each analysis was repeated twice (biological replicates) with three replicates in each experiment (technical replicates).

### CG MD simulations

CG MD simulations were conducted using the Martini v2.1 force field and GROMACS v5.0.7 (*61*, *62*). A structural model of the core domains of PLCγ1 (nPH, EF hands, TIM, and C2) was obtained from the autoinhibited structure by removing the coordinates for the sPH, nSH2, cSH3, and SH3 domains and remodeling missing atoms in loop regions using the program PRIME in the Schrodinger suite. The model used for CG simulations comprises residues 22 to 488 and 934 to 1215. A model of clone E was obtained by mutating residue 41, 42, 45, 166, 175, 195, 204, and 990 to alanine using MODELLER (*63*).

The WT core and the core^E^ (incorporating eight amino acid substitutions to alanine) models were converted to CG representations for the martini v2.1 force field using the martinize tool provided by the martini developers (*64*). Each CG protein was placed in a cubic periodic box of dimensions 21 nm by 21 nm by 26 nm, and energy minimized using the steepest descent method. The insane tool for computational lipidomics was used to construct a symmetric model membrane (composition: 40% POPE, 25% CHOL, 15% POPS, 10% POPC, 7% POP2, and 3% POP3), solubilize, and neutralize the system with Martini waters, sodium, and chloride ions (*65*). The system was energy-minimized using the steepest descent method and subjected to three rounds of equilibration in the NPT ensemble: for 20 ns with 5-fs time step, 1.5 ns with 7.5-fs time step, and then 2 ns with 10-fs time step. For all equilibrations, we used the velocity-rescaling thermostat at 323 K and the semi-isotropic Parrinello-Rahman barostat at 1 bar, and the protein backbone particles were restrained (*66*, *67*). Production simulations were run for 3 μs with 20-fs time step, using the velocity-rescaling thermostat (323 K) and semi-isotropic Parrinello-Rahman barostat (1 bar). Forty replicate production simulations for each protein were initialized from the equilibrated system, with velocities sampled from a Boltzmann distribution.

For simulations without the TIM barrel/sPH loops, WT core simulation replicate 39 was selected for extended simulation. The loop residues (471 to 488 and 934 to 947; sequence AYEEVPTSVMYSENDISN and DARLTEGKMMERRK) and two sodium ions (to balance charge) were removed from the system, and an additional 1 μs of simulation was conducted.

Simulations of the sPH, nSH2-cSH2, and SH3 domain structures were conducted and analyzed using the same methods as for the PLCγ1 core, with adjustments due to the smaller protein sizes (simulation time: 1 μs; number of replicates: 20; box dimensions: 16.5 nm by 16.5 nm by 20.5 nm).

### Atomistic MD simulations

Conversion to an atomistic representation for the CHARMM36 force field was achieved using the backward method (*68*, *69*). H335 and H380 were protonated, and a calcium ion was placed in the active site at the position observed in the crystal structure. The latest CHARMM36 lipid parameters were obtained from CHARMMGUI, with the Martini to CHARMM36 lipid type mappings described in table S2 (*70*). POPI25 was chosen as a model for PI(4,5)P_2_ on the basis of ab initio studies, indicating that this is the preferred protonation state (*71*). The backmapped protein coordinates were replaced with those from the original atomistic PDB after superimposition upon the backmapped structure, to correct any structural changes occurring during CG simulation and backmapping. To ensure the correct headgroup stereochemistry of the active site PIP_2_ after backmapping, the headgroup coordinates of this POPI25 were replaced by superimposition with those of a reference POPI25 obtained from a pure POPI35 membrane constructed using the CHARMM-GUI membrane builder (*70*). The backmapped protein and lipids were placed in a 19.6 nm by 19.6 nm by 16.5 nm cubic periodic box, energy-minimized (steepest descent), and subjected to 1 ns of equilibration with a 1-fs time step in the NPT ensemble, as described for the CG equilibrations. An unrestrained production simulation was run for 1 μs, with a 2-fs time step, a temperature of 323 K, and a semi-isotropic Parrinello-Rahman pressure coupling at 1 bar (*66*, *67*).

For restrained MD simulations to further investigate the geometry of PI(4,5)P_2_ in the active site, harmonic distance restraints were applied between the following atoms of the bound PIP_2_ and the protein: PIP_2_:P1-HIS380:NE2; PIP_2_:P1-HIS335:NE2; PIP_2_:P4-LYS462:NZ; and PIP_2_:O3-ARG988:CZ. Restraints were based on the distances observed in the IP3-bound crystal structure and are detailed in Supplementary Text. The restrained simulation was initiated from the end point of the 1-μs unrestrained atomistic simulation and simulated for 10 ns, keeping all other settings the same.

### Simulation analysis

For analysis, the protein was centered in the simulation box throughout the trajectories to prevent artifacts arising due to periodic boundary conditions (PBC). Root mean square deviation and root mean square fluctuation of protein backbone particles were calculated for each trajectory relative to its first frame. The GROMACS dist module was used to calculate the *z*-axis distance between the protein and membrane centers of mass. The protein orientation was determined by using the GROMACS gangle module to measure the angle (θ) between the plane defined by the backbone particles of residues 416, 1011, and 1194 and the *z* axis of the simulation box. The purpose of distance and orientation analysis was to understand the geometry of the protein relative to membrane, and hence, the measurements were corrected to account for PBC, which allowed the protein to bind to either leaflet of the membrane by traveling through the periodic boundary on the *z* axis. PBC corrections were applied using the following equations for all simulation frames where *z*-dist_uncorrected_ < 0 (i.e., when the protein was below the membrane center of mass): θ = 180° − θ_uncorrected_ and *z*-dist = −1* *z*-dist_uncorrected_. Corrections were performed using Python before further analysis and visualization.

The distance-orientation density plot was generated as a two-dimensional histogram of *z*-distance and θ over all simulation frames. Particle *z*-axis density plots were constructed using gmx density, taking the symmetrized density along the *z* axis relative to the membrane center of mass for the following particle groups: phospholipid tails, phospholipid headgroups, C2 hydrophobic loop, TIM hydrophobic ridge, and protein. To ensure that this *z*-axis density analysis characterized only the identified preferred orientation of the core, it was conducted only for simulations that ended with a θ between 6° and 12° and protein-membrane *z*-axis distance < 4.5 nm and only for the final 500 ns of these simulations.

Contacts between all residues and all lipid particles were calculated using the following groupings for lipids: POPC headgroup, POPE headgroup, POPS headgroup, POP2 headgroup, POP3 headgroup, and CHOL. Using the GROMACS mindist module, we determined whether a molecule had particles belonging to the given lipid group and that were within a 5.5 Å cutoff distance of any particles belonging to each protein residue. For each lipid molecule satisfying this criterion during a simulation frame, one contact was counted. Contacts were totalled over all frames during the final 1000 ns of each simulation, for each residue and lipid group. Normalized frequency of contacts was obtained by dividing the total contacts at each residue by the total contacts made by the residue with the highest number of contacts within that lipid group. Thus, the residue with the highest number of contacts has the normalized value 1, and the contacts for all other residues are normalized relative to this residue. Convergence analysis was carried out as above, using differently sized samples of simulation replicates.

### Bioinformatic analysis

Structural alignments were conducted in VMD, using the STAMP method (*72*, *73*). Sequence alignments were conducted in JalView, using the ClustalW method and canonical human PLC sequences obtained from UniProt (PLCγ1: P19174; PLCδ1: P51178; PLCζ1: Q86YW0) (*74*).

## Notes

View/request a protocol for this paper from *Bio-protocol*.
