# Supplementary material for: Characterization of the membrane interactions of phospholipase Cγ reveals key features of the active enzyme
Source: Sci Adv. 2022 Jun 24;8(25):eabp9688. doi: 10.1126/sciadv.abp9688 (PMC9232102; doi:10.1126/sciadv.abp9688)
Supplement: Supplementary file 1 — Figs. S1 to S14 Tables S1 and S2 Supplementary text [file sciadv.abp9688_sm.pdf]

Science Advances
AAAS

# Supplementary Materials for

## Characterization of the membrane interactions of phospholipase Cγ reveals key features of the active enzyme

Kyle I. P. Le Huray *et al.*

Corresponding author: Tom D. Bunney, t.bunney@ucl.ac.uk; Antreas C. Kalli, a.kalli@leeds.ac.uk; Matilda Katan, m.katan@ucl.ac.uk

*Sci. Adv.* **8**, eabp9688 (2022)
DOI: 10.1126/sciadv.abp9688

**This PDF file includes:**

Figs. S1 to S14
Tables S1 and S2
Supplementary text

**Fig. S1.**

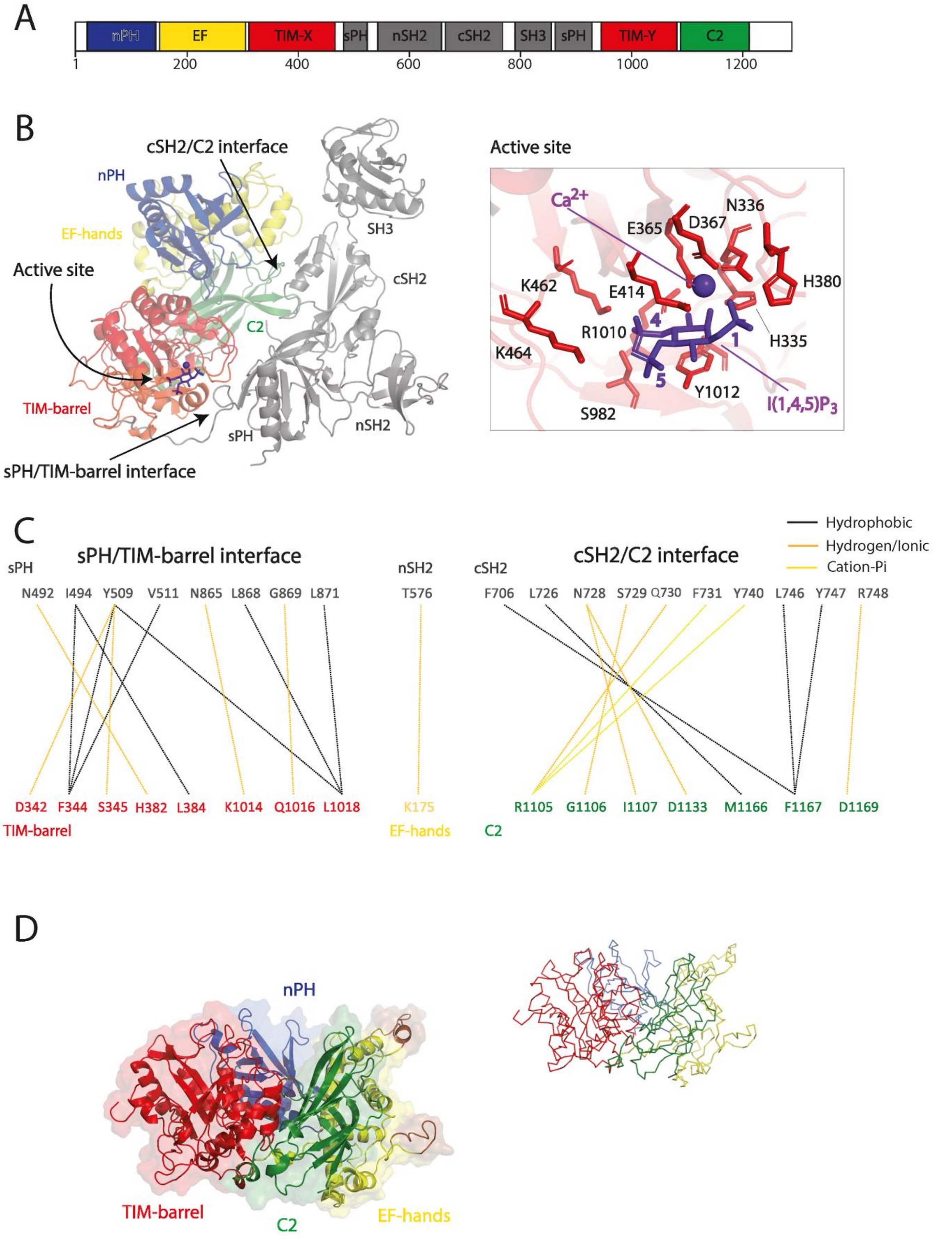


**Fig. S1. PLCγ1 structure and features of the autoinhibitory interfaces and the PLC-core. (A)** Schematic diagram of PLCγ1, with residue numbers indicated in the linear sequence. The

individual domains of the γSA are in grey, and the PLC-core domains coloured individually (TIM-barrel: red, nPH: blue, EF-hands: yellow, C2: green). In the crystal structure of the protein (including amino acids 21-765 and 791-1215, joined by the SGS linker) (PDB ID: 7Z3J) only a few segments are disordered: 190-206, 234-244, 524-543 and 935-946. **(B)** PLCγ1 structure (PDB ID: 7Z3J) with the domains of the γSA and PLC-core coloured as in part A. The view depicts the active site, with bound calcium cofactor and $IP_3$, and two autoinhibitory interfaces (left panel). In the expanded view of the active site (right panel), the side chains of residues (red) interacting with calcium (purple sphere) and $IP_3$ (inositol 1,4,5 structure in purple) are shown and labelled; positions of 1, 4 and 5 phosphates in $IP_3$ are also indicated. The relative positions and distances between the side chains and calcium/$IP_3$ are essentially the same as in PLCδ1 (PDB ID: 1DJX). **(C)** Interactions of the key residues in the interfaces are represented as a diagram. The interfaces of the sPH/TIM-barrel and cSH2/C2 are quite extensive; the interaction between the nSH2 and EF-hands includes one weak interaction, with the K175 side chain positioned slightly differently in two crystal structures (PDB ID: 7Z3J and PDB ID: 6PBC). **(D)** Side view of the PLC-core showing the TIM-barrel, nPH, C2 and EF-hands. The loops not visible in crystal structures of rat PLCγ1, indicated in brown in EF-hands, are derived from a model (prepared in Schrodinger suite by the program Prime) and are included in the PLC-core (ribbon, right panel) used in further analyses. Since rat and human PLCγ1 have 98% identity, a replacement of non-identical amino acids from human to rat sequence is without significant changes in the protein structure. Consequently, the overall structure and that of the PLC-core shown here is representative for rat and human proteins.

**Fig. S2.**

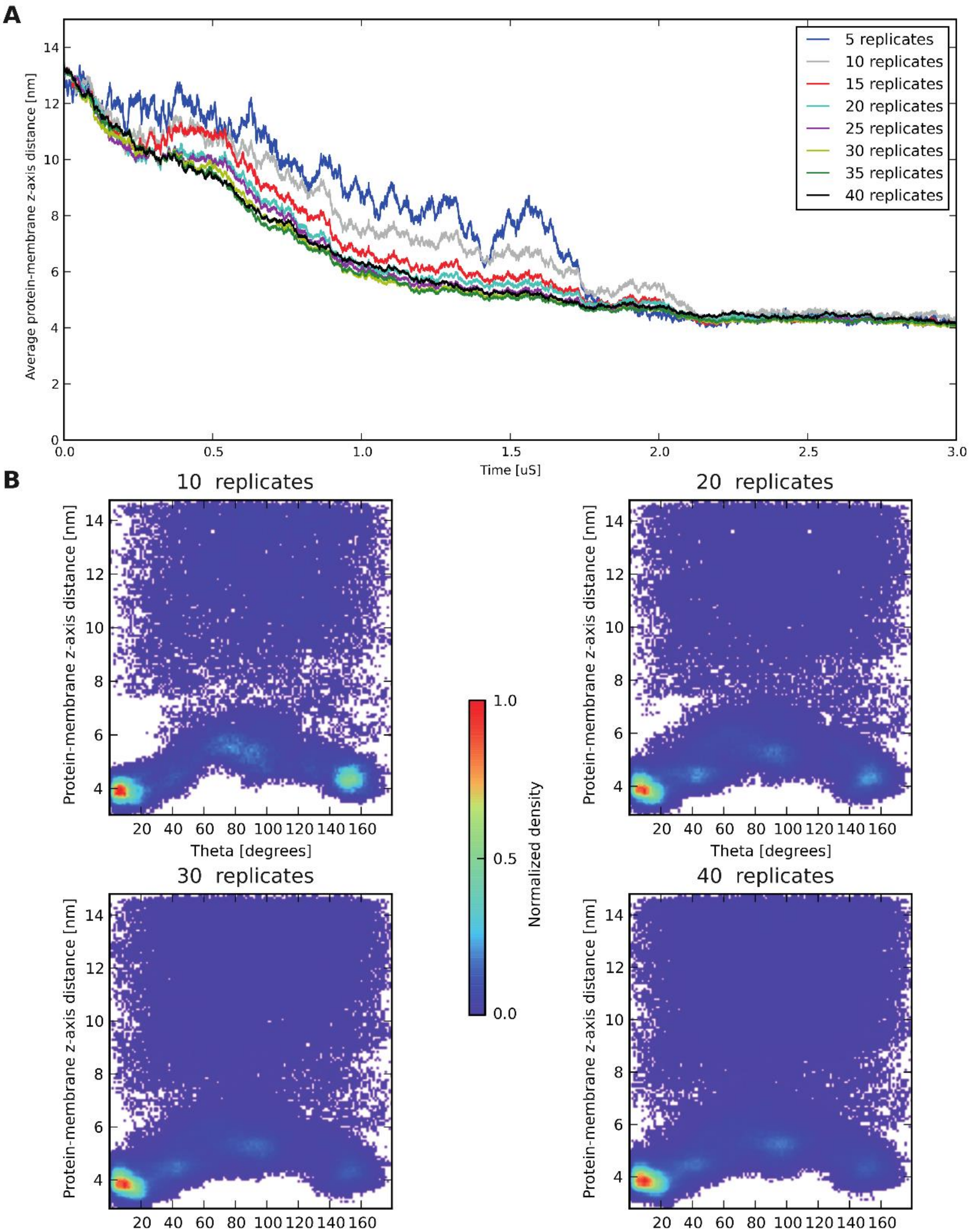


**Fig. S2. Simulation data converge with 40 independent replicates. (A)** mean z-axis distance between protein and membrane centres of mass over 3 µs of CG MD simulation. Coloured traces show the mean calculated from increasing sample sizes of simulation replicates. Forty independent simulations (black trace) provide sufficient sampling for convergence. **(B)** 2D histograms of protein-membrane z-axis distance and orientation (quantified by theta, see methods) showing the distance-orientation landscape sampled during simulations. Histograms calculated for 10, 20, 30

and 40 simulation replicates. Forty replicates provide excellent sampling of the landscape and identification of the global and local minima on the membrane.

**Fig. S3.**

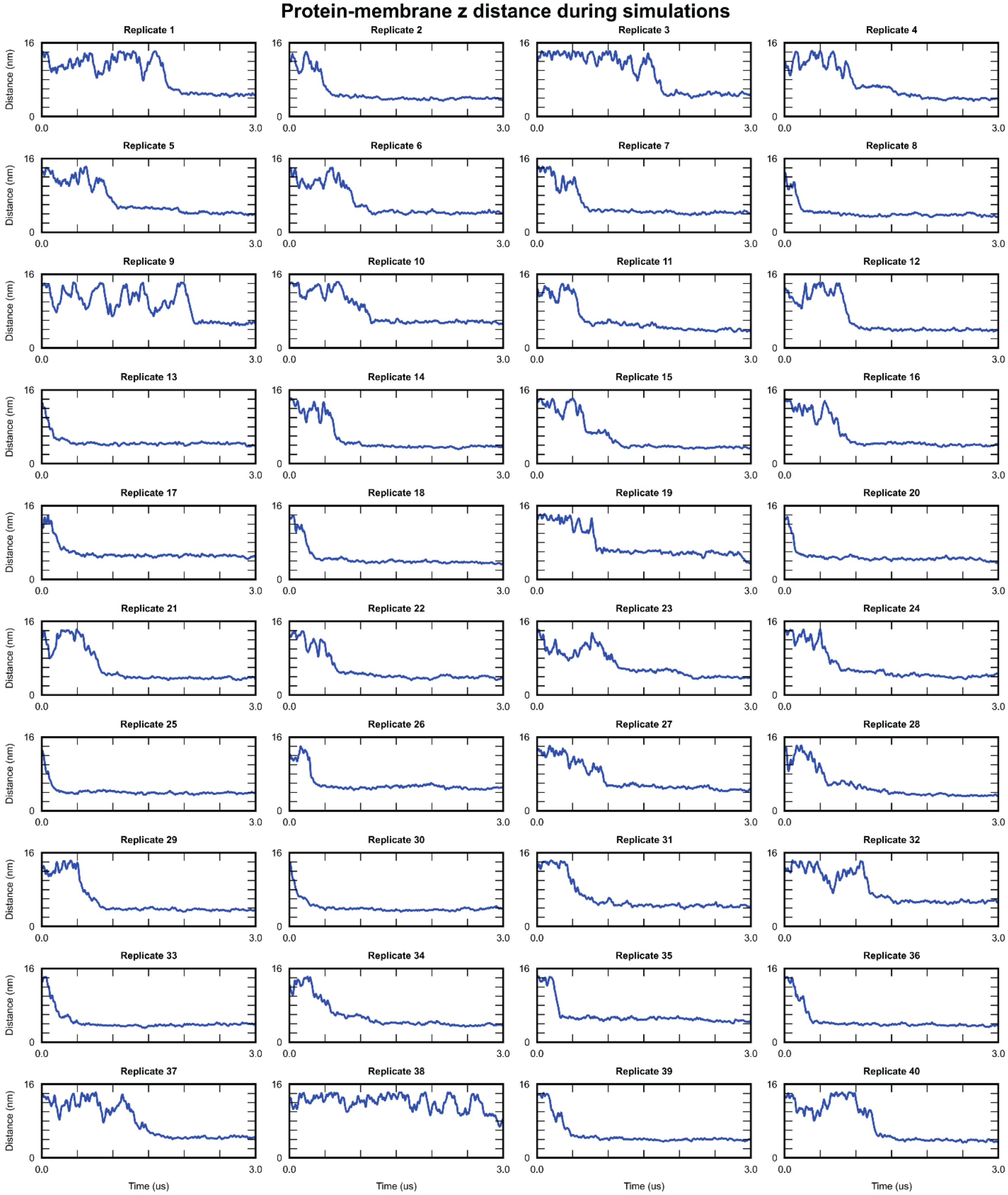


**Fig. S3. Protein-membrane distance for all simulations.** The z-axis distance (nm) between the protein and membrane centres of mass (50 frame running average) is shown for each simulation replicate and has been corrected to account for periodic boundary conditions. Membrane binding occurs at a distance of approximately 4-5 nm, and stable membrane binding is observed in 39/40

replicates. Dissociation events were not observed following stable binding. Replicates are independent due to randomization of initial particle velocities.

**Fig. S4.**

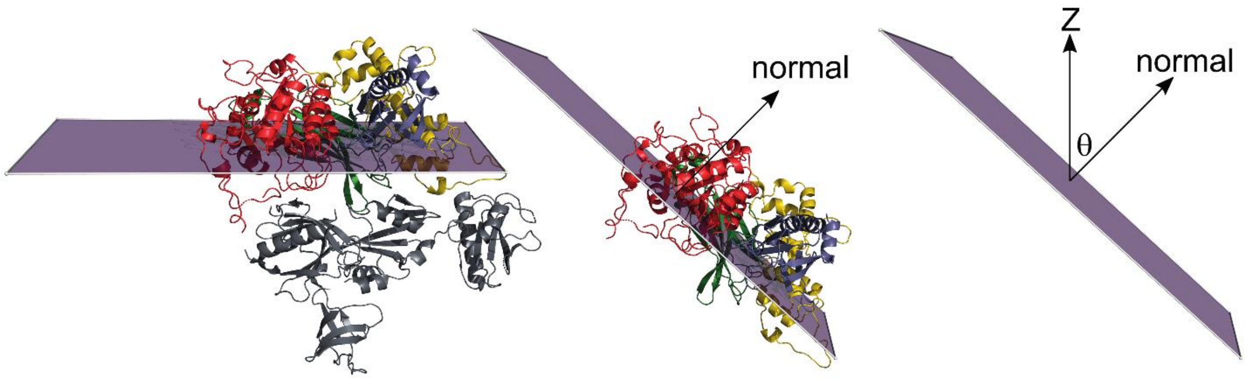


**Fig. S4. Quantifying the orientation of the PLCγ1 core relative to the membrane.** The backbone particles of residues 416, 1011 and 1194 were selected to define a plane, and the angle θ between the normal of this plane and the z-axis of the simulation box (corrected for periodicity) was measured for all simulation frames. The selected residues are in structured regions of the protein, and due to the elastic restraint network employed in the Martini force field, it is acceptable to treat their relative positions as constant for the purpose of this analysis. The membrane, on average, lies along the XY-plane of the simulation box, and the residues were selected such that they define a plane which lies close to the plane of autoinhibition. Therefore, if the PLCγ1 core binds to the membrane with the same interfaces used for autoinhibition, then a preferred membrane bound state with low value of θ is expected.

**Fig. S5.**

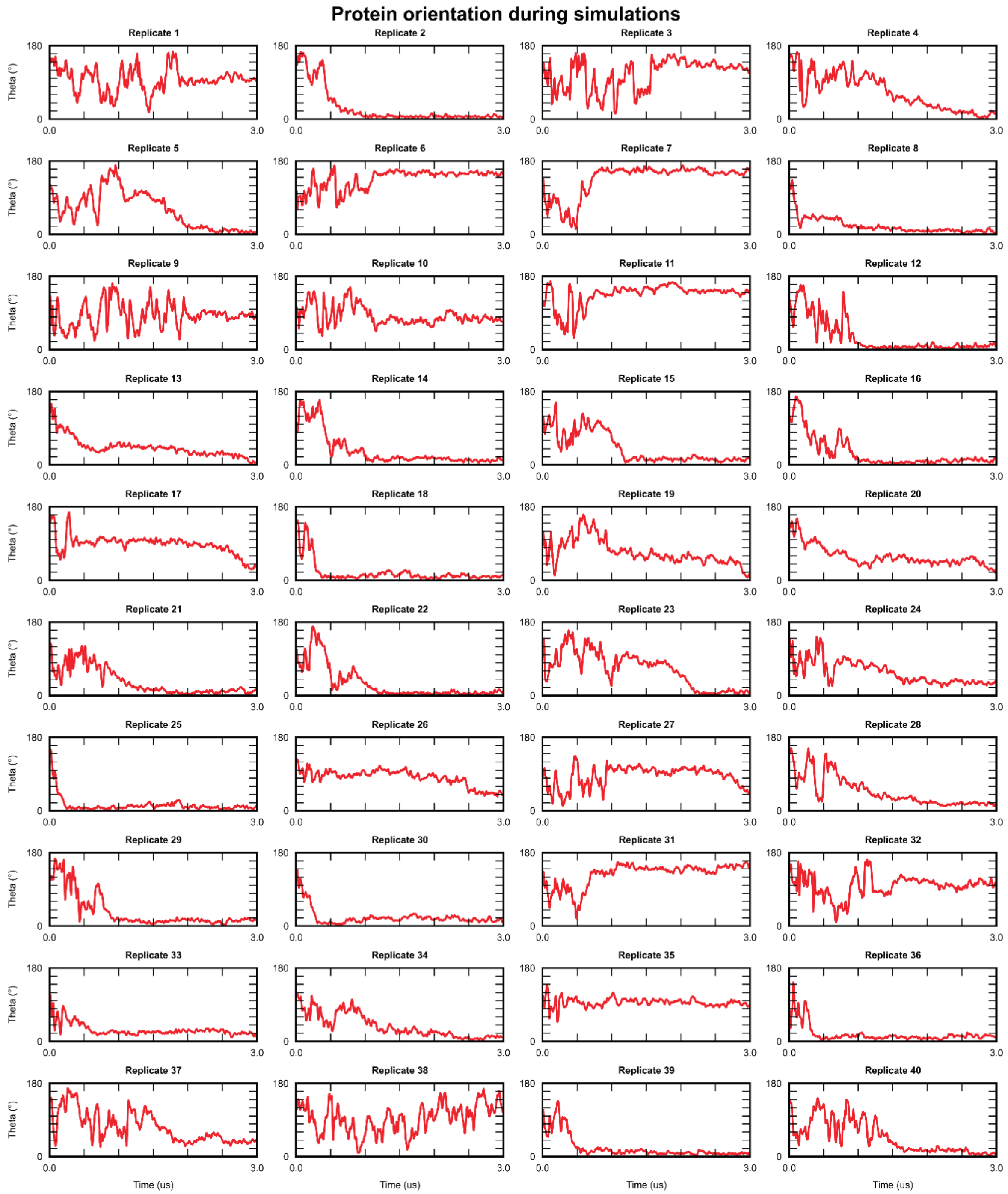


**Fig. S5. Orientation of the PLCγ1 core over time for all simulations.** θ, defined as the angle between the simulation box Z-axis and the normal to the plane formed by backbone particles of residues 416, 1011 and 1194 is plotted as a 50 frame running average for each simulation replicate. As the membrane, on average, lies on the XY-plane of the simulation box, θ provides a measure of the orientation of the PLCγ1 core relative to the membrane. θ has been corrected to account for periodic boundary conditions. Although all simulations start with the same initial geometry, the

PLCγ1 core freely tumbles in the solution before encountering the membrane. Replicates are furthermore independent due to randomization of initial particle velocities. The initial orientation therefore does not bias the sampling of membrane bound states. The PLCγ1 core samples many orientations on the membrane, with a global minimum at $\theta \approx 9°$.

**Fig. S6**

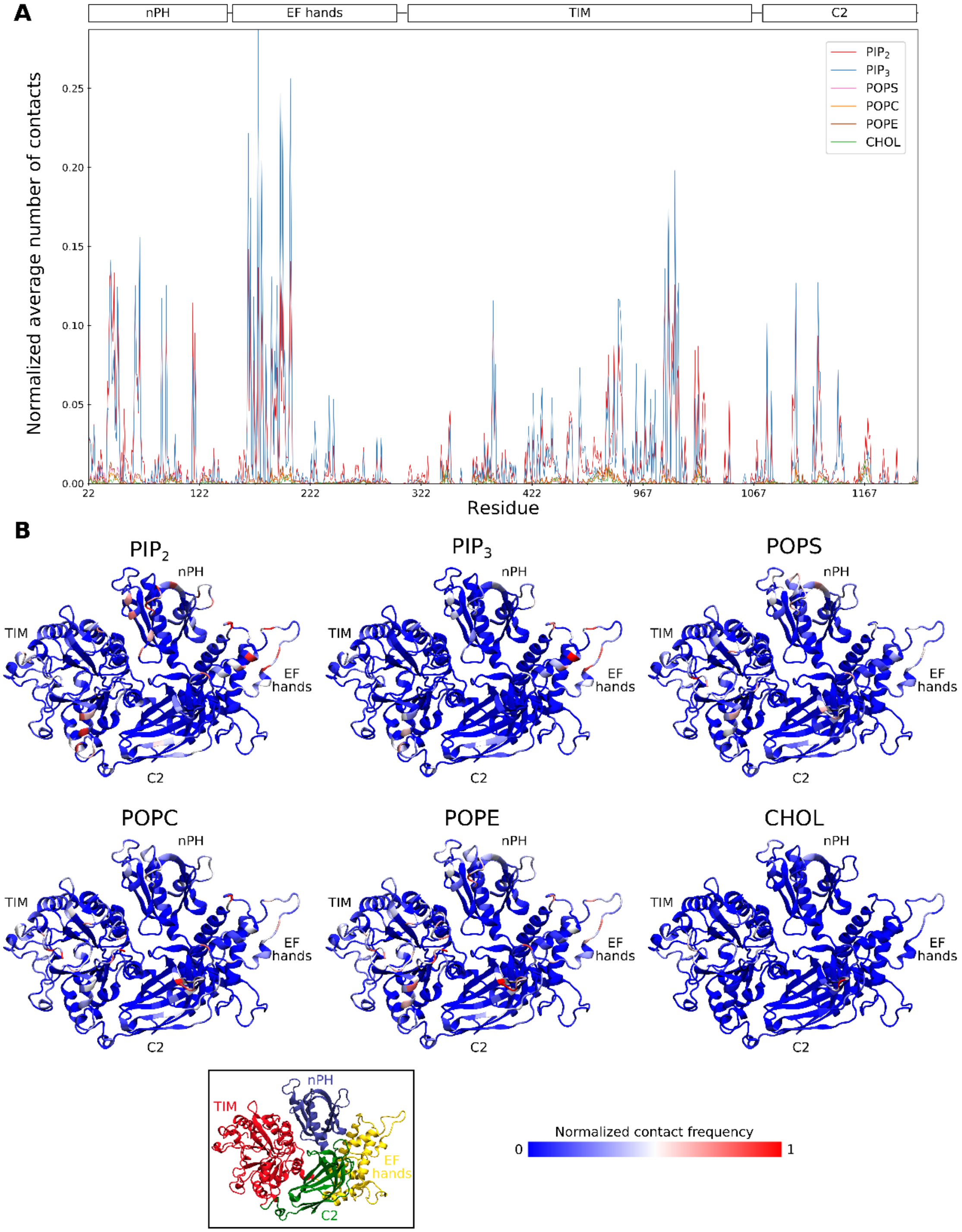


**Fig. S6. Lipid interaction hotspots of the PLCγ1 core. (A)** Normalized average number of contacts between each residue of the PLCγ1 core and different lipid types in the simulation system ($PIP_2$ headgroup: red, $PIP_3$ headgroup: blue, POPS headgroup: pink, POPC headgroup: orange, POPE headgroup: brown, CHOL: green). Normalization here was conducted by dividing the total

contacts by the number of simulation frames and the number of each lipid species. This analysis therefore reflects preferences for interaction with different lipid species at each residue. **(B)** Normalized frequency of contacts between each residue of the PLCγ1 core and different lipid species in the simulation, visualized on the PLCγ1 core structure (inset: PLCγ1 core structure colored by domain). Normalization here was conducted by dividing the total contacts at each residue by the total contacts of the residue which had the most contacts with the given lipid species. Therefore, the residue which had the most contacts with a particular lipid species has the value 1 (most red), and the contacts for all other residues are scaled accordingly.

**Fig. S7**

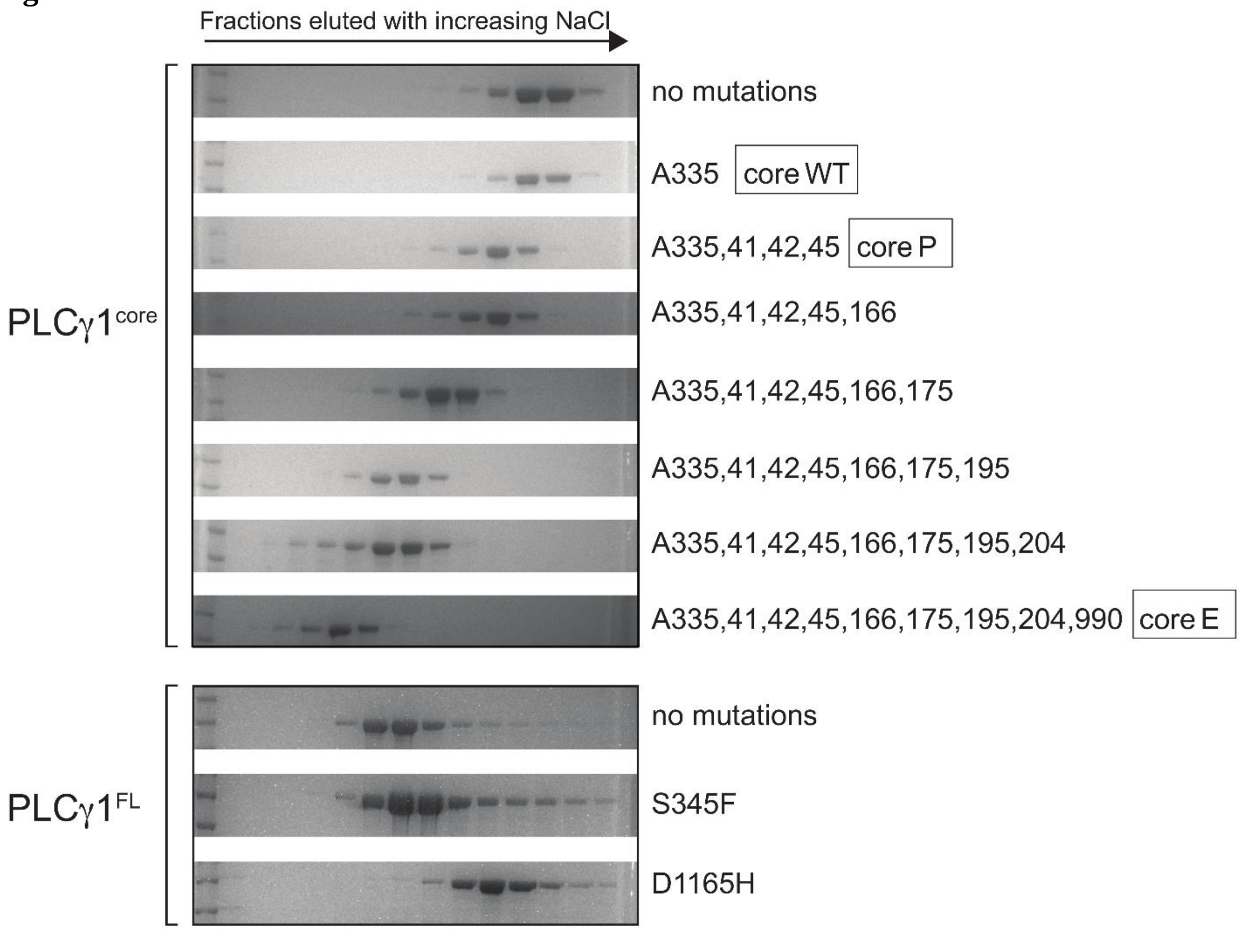


**Fig. S7. The Heparin Sepharose mobility of various PLCγ1 constructs.** The indicated core and full length PLCγ1 variants were all purified using Heparin Sepharose chromatography. Each of the core constructs incorporated alanine substitutions at the positions (numbered) of arginine or lysine residues implicated in membrane interactions: K41, K42 and R45 in the nPH domain, R166, K175, R195 and R204 in the EF-hands and K990 in the TIM-barrel domain. Additionally, the catalytic histidine (H335) was also replaced by alanine in these constructs to allow liposome binding, when containing $PIP_2$. The designations, core WT, core P and core E, are shown for the corresponding variants with specified mutations. The core variants each eluted at a different NaCl concentration, with clear correlation between the number of mutations removing arginine and lysine residues and the decreasing affinity for heparin. For the full-length proteins, mutations that lead to an activated enzyme had increased affinity for heparin.

**Fig. S8**

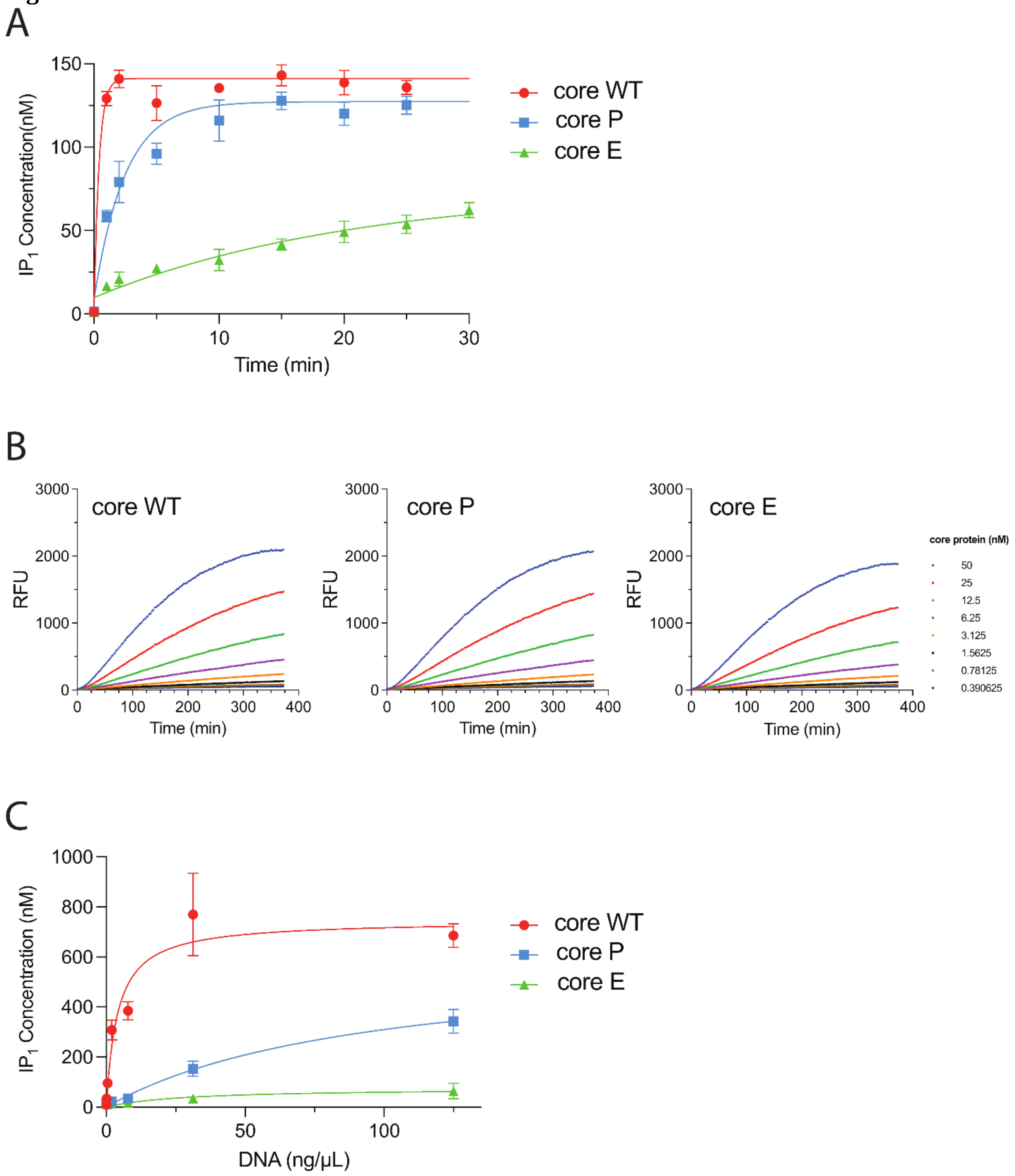


**Fig. S8. The identified lipid interaction sites are critical for catalytic activity of PLCγ1 on membranes and in cells but not on soluble substrate. (A)** The rate of hydrolysis of phosphatidylinositol incorporated in liposomes by the PLCγ1$^{core}$ constructs (designated as core WT, core P and core E) at a final enzyme concentration of 50 nM. The quantity of $IP_1$ produced was measured at multiple timepoints using HTRF with the addition of a labelled $IP_1$ probe and an anti-$IP_1$ labelled cryptate antibody. **(B)** Enzyme progress curves showing the rate of hydrolysis of

the soluble substrate Aldol 518 myo-inositol-1-phosphate at 25 mM by the PLCγ1$^{core}$ constructs at the indicated enzyme concentrations, monitored by measuring fluorescence of the reaction product. **(C)** The quantity of $IP_1$ generated in HEK293 cells lysed 49 hours after transfection with varying amounts of plasmid expressing eGFP tagged PLCγ1$^{core}$ variants (core WT, core P and core E). $IP_1$ produced was quantified using the HTRF assay.

**Fig. S9**

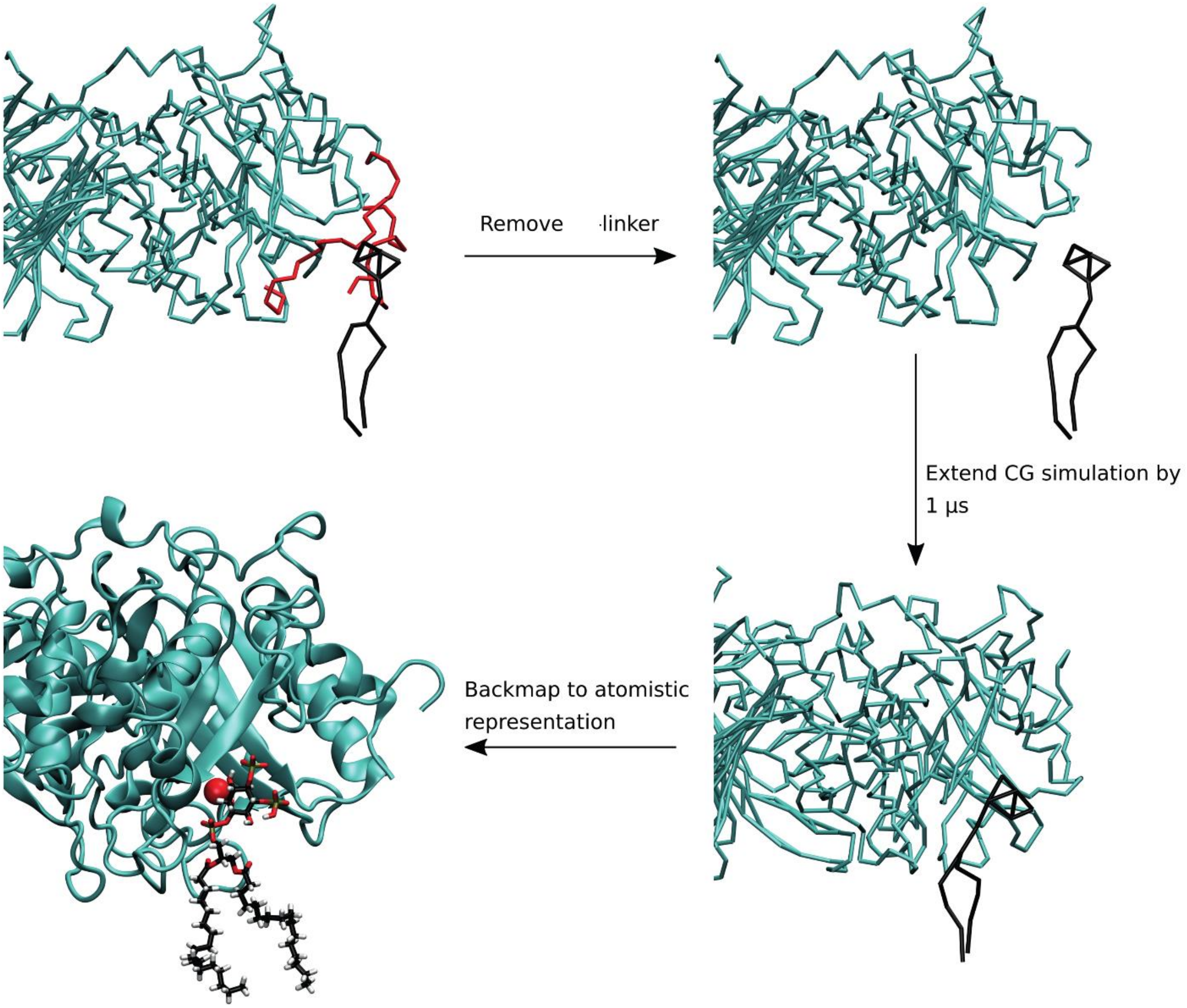


**Fig. S9. Removal of the TIM-barrel/sPH linkers and backmapping from coarse-grained to atomistic representation.** After 1 μs of CG simulation, $PIP_2$ (black sticks) was found to be in the vicinity of the PLCγ1 (cyan) active site but obstructed by the TIM-barrel/sPH linker loops (red). To facilitate $PIP_2$ entry to the active site, the linker loop regions were removed and a further 1 μs of CG simulation was conducted. During this extended simulation, $PIP_2$ moved into the active site. The end point of the extended simulation was backmapped to an all-atom representation (see methods), as the starting point for further atomistic simulation.

**Fig. S10**

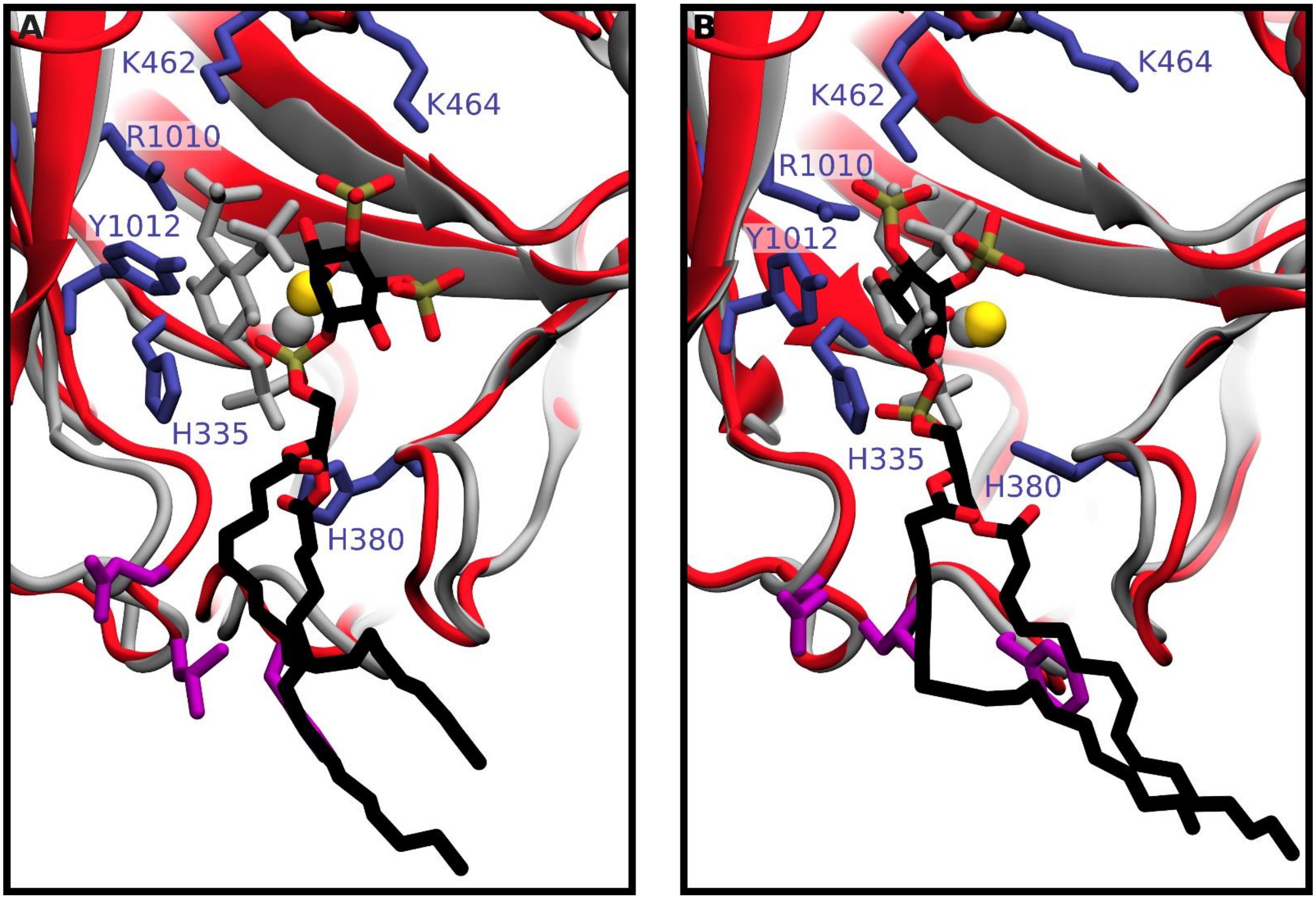


**Fig. S10. Comparison of simulated and crystallographic ligand binding to the PLCγ1 active site.** STAMP structure alignment of simulated PLCγ1 with PI(4,5)$P_2$ at the active site, and the crystal structure with $IP_3$ at the active site. **(A)** position of the bound $PIP_2$ observed during the unrestrained atomistic MD simulation and **(B)** position of the bound $PIP_2$ after distant restraints were applied to the headgroup based on the crystallographic evidence. The protein backbone is shown as a ribbon diagram (simulation: red, crystal: grey), ligands are shown as sticks (simulated PIP2: colored by element, crystallographic IP3: grey) and the $Ca^{2+}$ cofactor as a sphere (simulation: yellow, crystal: grey). The sidechains of key protein residues around the active site are shown as blue sticks (simulation positions only) and hydrophobic residues F344, L384 and L1018 in the ridge which interact with the lipid acyl tail are shown as purple sticks (simulation positions only).

**Fig. S11**

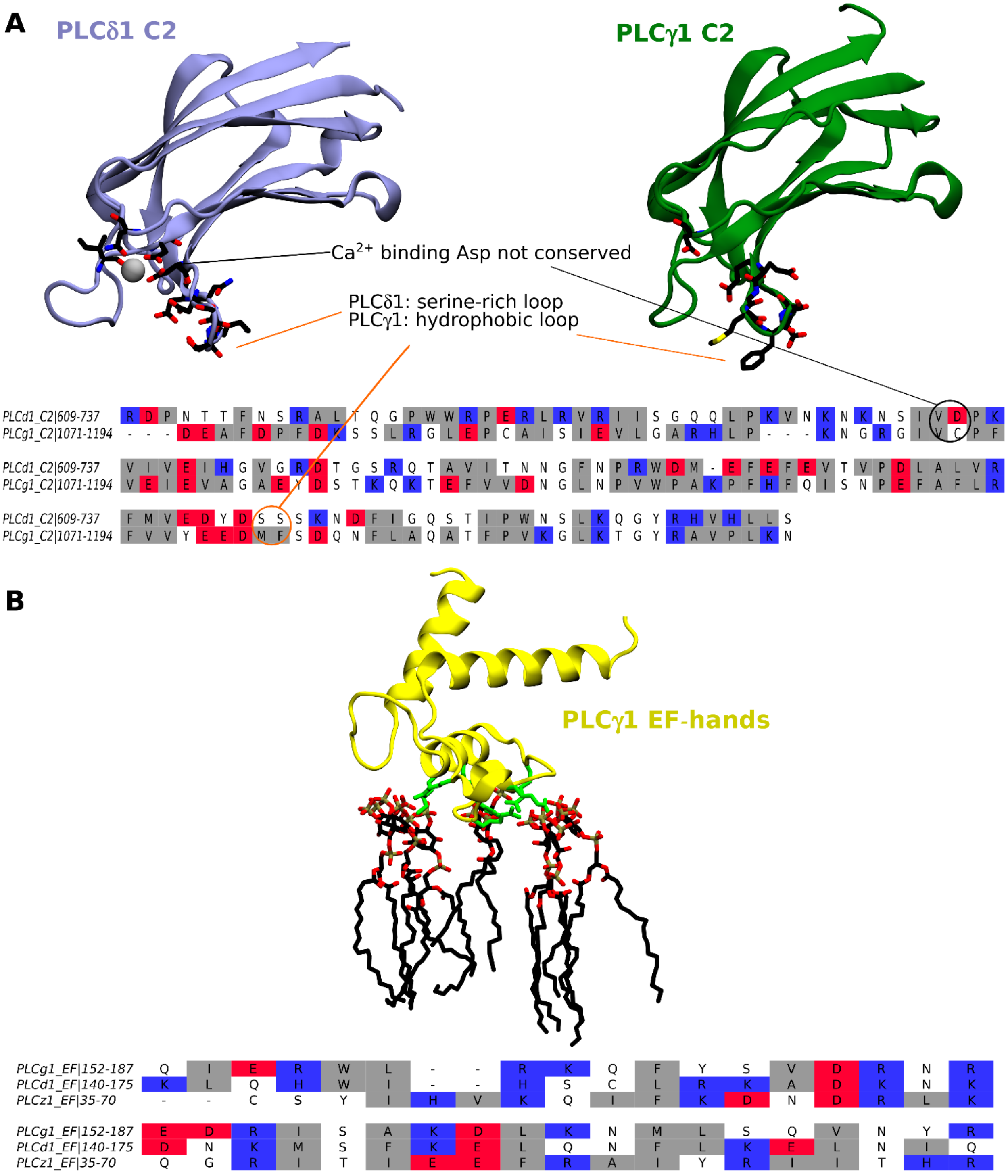


**Fig. S11. Comparison of PLCδ1 and PLCγ1 C2 domains, and detail of the lipid interactions of the PLCγ1 EF-hands. (A)** The structures of the PLCδ1 (PDB ID: 1DJX, purple, with bound $Ca^{2+}$) and PLCγ1 (PDB ID: 7Z3J, green) C2 domains, aligned structurally (top) using STAMP alignment, and sequentially (bottom) using ClustalW alignment. PLCγ1 appears to have lost the

key asparagine involved in coordinating $Ca^{2+}$ and has replaced the PLCδ1 serine-rich loop with a hydrophobic moiety. **(B)** Atomistic simulation snapshot (top) revealing detail of the PLCγ1 EF-hands associated with multiple anionic lipids through electrostatic interactions with a cluster of lysine and arginine residues (green sticks) situated at the membrane surface. ClustalW sequence alignment (bottom) of the PLCγ1, PLCδ1 and PLCζ1 EF-hand domains, highlighting sequence similarity.

**Fig. S12**

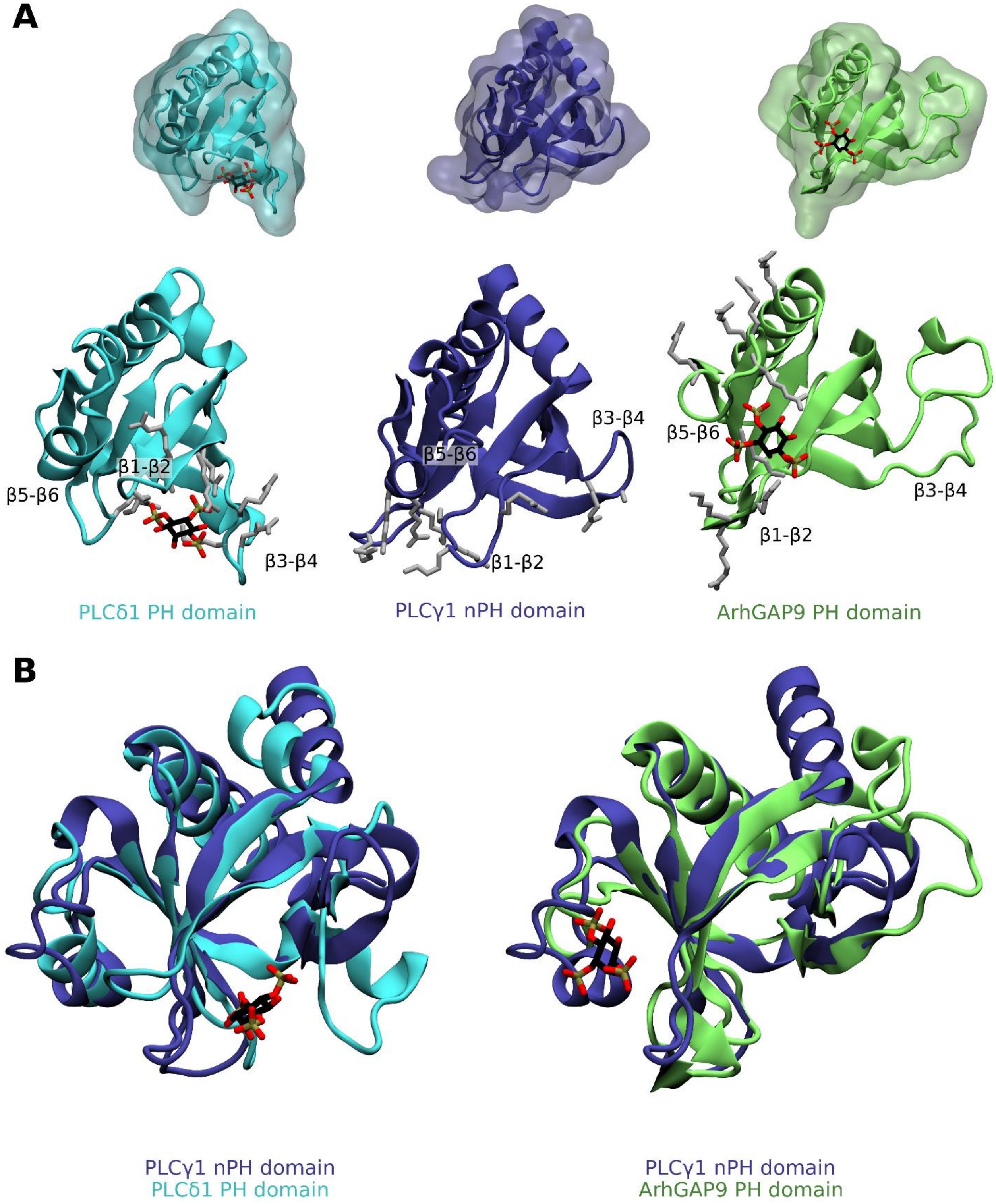


**Fig. S12. Comparison of phosphoinositide binding sites in the PLCδ1, PLCγ1 and ArhGAP9 PH domains.** Structures of the PLCδ1 PH domain (PDB ID: 1MAI, cyan), PLCγ1 nPH domain (PDB ID: 7Z3J, blue) and the ArhGAP9 PH domain (PDB ID: 2P0H, green). Bound inositol

trisphosphate observed in the PLCδ1 and ArhGAP9 structures is shown. **(A)** surface (upper panel) and ribbon (lower panel) representations of the three PH domains. Residues predicted by previously reported simulations to be likely sites of phosphoinositide interaction are shown as white sticks (*34*). The PLCδ1 PH domain structure demonstrates the canonical phosphoinositide binding site for PH domains, whereas ArhGAP9 has a non-canonical site on the outside of the barrel between β1-β2 and β5-β6 loops. The predicted interaction sites from simulation are in good agreement with the crystal structures for these two PH domains. However, the PLCγ1 nPH domain lacks a number of key lysine and arginine residues present in the PLCδ1 canonical binding site. PLCγ1 nPH instead has a cluster of basic residues on the outer face of the β-barrel, in the vicinity of the β1-β2 and β5-β6 loops and likely interacts with phosphoinositides or other anionic lipids at these non-canonical sites, similar to ArhGAP9. **(B)** STAMP structure alignment of PLCγ1 nPH with PLCδ1 PH (left, alignment RMSD: 2.64 Å), and PLCγ1 nPH with ArhGAP9 PH (right, alignment RMSD: 2.19 Å).

**Fig. S13**

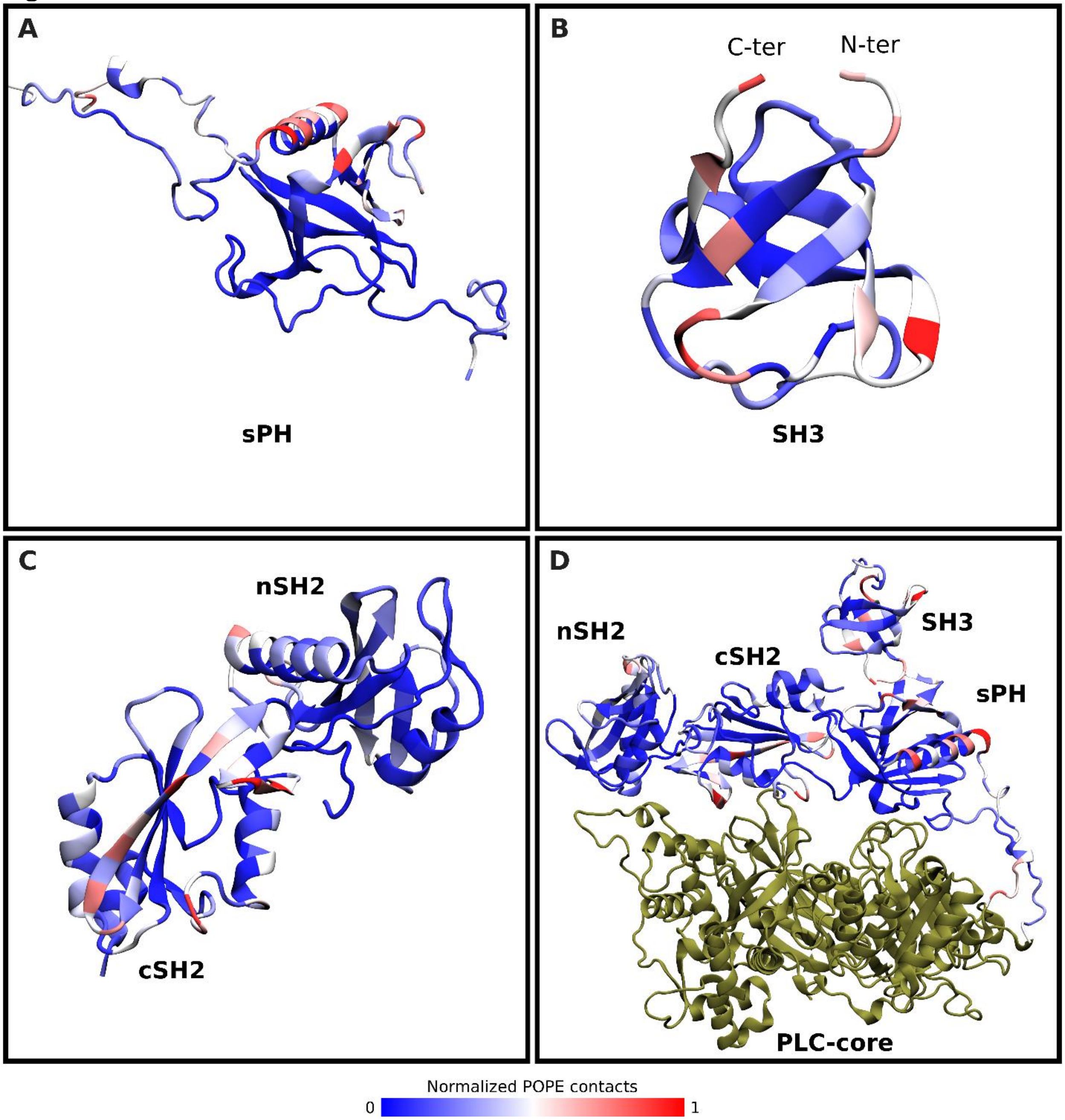


**Fig. S13. Predicted membrane interaction interfaces of γSA domains.** Structures of the PLCγ1 **(A)** sPH domain, **(B)** tandem nSH2-cSH2 domains, and **(C)** SH3 domain. Together these form the γSA. Each residue is coloured according to the normalized frequency of contacts with POPE headgroups during 20 independent 1 μs simulations for each structure. Normalization was conducted for each structure, such that the residue with the value 1 had the most contacts for that structure. **(D)** Shows the results for the structures in panels **(A-C)** in the context of full-length PLCγ1, with the core domains coloured gold and the γSA domains coloured as in panels **(A-C)**. The hotspots of POPE contact suggest possible interfaces for membrane interaction. This analysis does not give insight into the overall strength of membrane association for each domain and does not take into consideration intramolecular protein-protein interactions between the domains which

may compete with membrane binding; rather it predicts the most plausible regions of membrane interaction if membrane interaction occurs. For the $cSH_2$ domain, membrane interactions were localized around its phosphotyrosine binding site. As, in the active form, this region would be bound to the segment of cSH2/SH3 linker surrounding pY783, and therefore it is not likely to be a physiological site of membrane interaction

**Fig. S14**

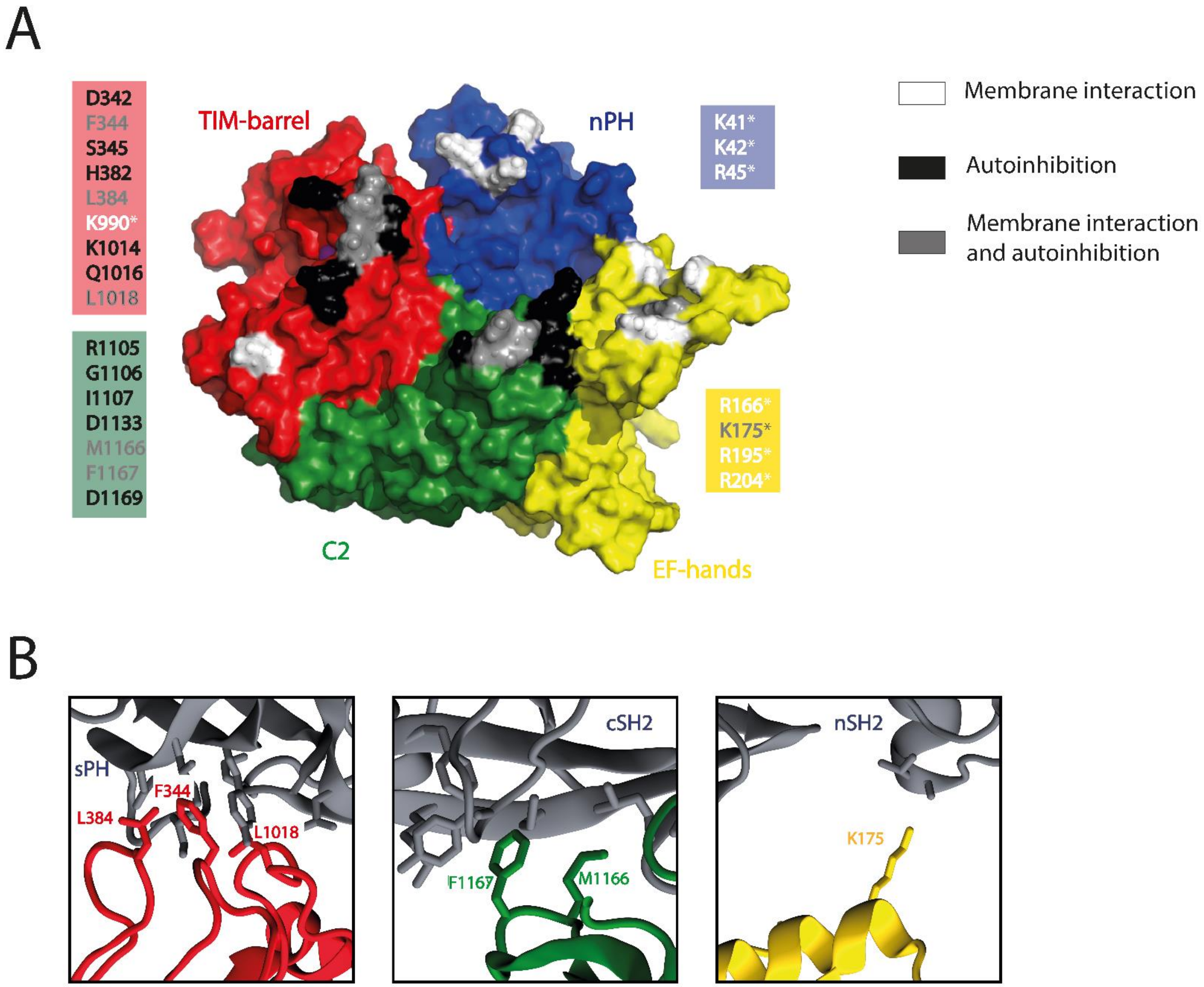


**Fig. S14. Comparison of the membrane interaction surfaces and autoinhibitory interfaces on the PLC-core (A)** Top view of the PLC-core (TIM-barrel: red, nPH: blue, EF-hands: yellow, C2: green). Residues involved in membrane interactions, autoinhibitory contacts or both in each of the PLC-core domains are shown and listed in white, black, and grey, respectively. Residues mutated in this study are indicated by asterisks (*). **(B)** Residues common for membrane interactions and autoinhibitory contacts in the TIM-barrel, C2 domain and EF-hands are shown in the structure of an autoinhibited PLCγ1.

**Table S1.**

| | rPLCγ1 construct |
|---|---|
| **Data Collection** | |
| Beamline | ID23-1 (ESRF) |
| Wavelength (Å) | 0.88560 |
| Resolution Range (Å, deg) | 115.1 – 2.0 (2.05 – 2.00) |
| Space group | $P\ 2_1\ 2_1\ 2_1$ |
| Cell parameters a, b, c (Å) | 72.76, 82.44, 230.11 |
| Total reflections | 388,054 (28,825) |
| Unique reflections | 93,254 (6,834) |
| Multiplicity | 13.5 (13.6) |
| Completeness (%) | 98.8 (99.6) |
| Mean I/Sigma(I) | 10.83 (0.55) |
| Wilson B-factor (Å$^2$) | 51.38 |
| $R_{meas}$ (%) | 7.6 (271.9) |
| $CC_{1/2}$ | 0.999 (0.234) |
| **Refinement** | |
| $R_{work}/R_{free}$ (%) | 22.41/25.51 |
| Number of non-hydrogen atoms | 9,400 |
| Protein atoms | 9,113 |
| Solvent molecules | 243 |
| Ligands | 44 |
| Protein residues | 1120 |
| B-factor (Å$^2$) - average | 62.31 |
| Protein | 62.49 |
| Solvent | 55.57 |
| Ligands | 63.73 |
| Ramachandran Plot | |
| Favoured (%) | 94.95 |
| Allowed (%) | 4.60 |
| Outliers (%) | 0.45 |
| Rotamer outliers | 2.21 |
| Clash score | 5.64 |
| Rmsd | |
| Bonds (Å) | 0.0084 |
| Angles (deg) | 0.97 |
| PDB ID | 7Z3J |

**Table S1:** Crystallographic data collection and refinement statistics

**Table S2.**

| **MARTINI LIPID** | **CHARMM36 LIPID** |
| --- | --- |
| POPC | POPC |
| POPE | POPE |
| POPS | POPS |
| POP2 | POPI25 (= PI(4,5)$P_2$ with PO tail and proton on position 5 phosphate) |
| POP3 | POPI35 (= PI(3,4,5)$P_3$ with PO tail and proton on position 5 phosphate) |
| CHOL | CHL1 |

**Table S2**: Conversion from Martini v2.1 to CHARMM36 lipid types

**Supplementary Text**

Further methodological detail for restrained MD simulations

For restrained MD simulations to further investigate the geometry of PI(4,5)P2 in the active site, harmonic distance restraints were applied between the following atoms of the bound PIP2 and the protein: PIP2:P1--HIS380:NE2; PIP2:P1--HIS335:NE2; PIP2:P4--LYS462:NZ; and PIP2:O3--ARG988:CZ. Restraints were based on the distances observed in the IP3-bound crystal structure. The restrained simulation was initiated from the endpoint of the 1 μs unrestrained atomistic simulation and simulated for 10 ns, keeping all other settings the same.

The atom numbers in the simulation correspond to:

11654 = PIP2:P1
11654 = PIP2:P1
11636 = PIP2:P4
11631 = PIP2:O3
5895 = HIS380:NE2
5218 = HIS335:NE2
7219 = LYS462:NZ
8397 = ARG988:CZ

Distants restraints were thus applied by adding the following lines to the itp file containing the topology of the protein plus bound PIP2:

```
[ distance_restraints ]
; ai   aj   type  index  type'   low   up1   up2   fac
11654  5895  1     0      1      0.243  0.363  0.393  1.0
11654  5218  1     1      1      0.319  0.439  0.469  1.0
11636  7219  1     2      1      0.252  0.372  0.402  1.0
11631  8397  1     3      1      0.247  0.367  0.397  1.0
```
